# Influence of inner meshing profile on noise of hybrid electric vehicles coupler chain based on vibration analysis

**DOI:** 10.1038/s41598-025-05168-w

**Published:** 2025-07-01

**Authors:** Lichi An, Gongfan Zhang

**Affiliations:** 1 Department of Mechanical Engineering, Taiyuan Institute of Technology, Taiyuan, 030008 China; 2 School of Mechanical and Aerospace Engineering, Jilin University, Changchun, 130022 China

**Keywords:** HEVs coupler chain, Inner meshing profile, Vibration analysis, Chain noise, Wear analysis, Engineering, Mathematics and computing, Physics

## Abstract

Chain noise is an important factor affecting the ride comfort of hybrid electric vehicles (HEVs) with chain coupler. The inner meshing profile can effectively reduce the polygonal action, which has already been proved, but its influence on the noise is controversial. In this paper, the basic vibration locus, velocities, and acceleration for HEVs coupler chain are analyzed. Based on a specific example, under the action of the inner meshing profile, the indicators that represent the low, medium and high frequency vibration are proved to be reduced. Based on meshing relations, it is proved that the error of the inner meshing profile will cause the deviation of the chain pitch line and the meshing disorder, which may weaken the influence of inner meshing profile on reducing noise. Through conducting the dynamics simulation and the noise and wear experiment, the results show that the influence of the inner meshing profile on the noise has dual effect, and it can only reduce the noise after running-in. Moreover, under the action of the inner meshing profile, the noise level will be decreased with the increase of running time. This paper not only resolves the controversy but also provides an effective method for chain noise.

## Introduction

New energy vehicles(NEVs) can reduce greenhouse gas emissions by 80%, so it is an effective way to achieve the goal of carbon neutrality^1^. NEVs sold on the market mainly include: electric vehicles(EVs), hybrid electric vehicles(HEVs), and plug-in hybrid electric vehicles(PHEVs). Among them, HEVs are considered an effective intermediate step toward a zero-emission transportation system^2^. First of all, HEVs can save energy without supplementing with electricity which is most likely derived from thermal power generation^3^. Compared with internal combustion engine vehicles(ICEVs), HEVs can reduce the fuel consumption and pollutant emission by 50%^4^. Secondly, HEVs can not only take advantage of the high energy density of batterie at low speed, but also take advantage of the high power density of internal combustion engine at medium and high speed^5^. Thirdly, compared with the other types of NEVs, HEVs have a higher area of use because the battery is more affected by temperature. In some areas with extremely cold winters, such as Northern Japan, Northeast China, Russia, Northern Europe etc., other types of NEVs will not be able to start effectively in winter because of the battery feed^6^. Therefore, in the absence of the subsidies about EVs or electricity, HEVs should have better prospects for development. In the US, the proportion of HEVs sales increased from 8.6% in the first quarter to 9.6% in the second quarter, while that of other NEVs sales decreased^7^.

**Fig. 1:**
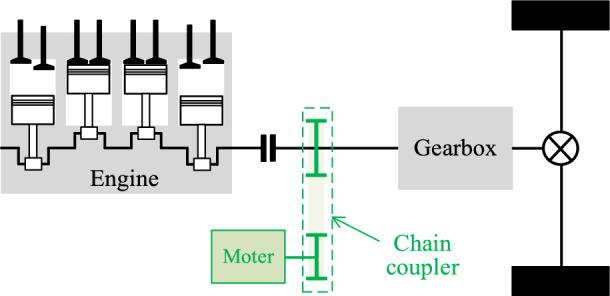
Chain coupler in vehicle.

According to the coupling object, HEVs coupling method can be divided into torque coupling, speed coupling, traction coupling, and complex coupling. According to the mechanical structure, torque coupling can be further divided into gear coupling, magnetic field coupling, chain/belt coupling^8^. Compared with gear coupling, chain coupling has the advantage of simple structure, small volume occupancy, light wight and easy control. In addition, it has better efficiency and NVH performance^9^. As Fig. 1 shows, the chain HEVs coupler is placed on one side of the vehicle, and couples torque from the engine and motor to the gearbox. Benefit from the advantages, an ICEV can be easily modified into a HEV by installing the chain HEVs coupler, so it is very suitable for the transformation and upgrading of the ICEVs manufacturers. The well-known chain coupler is the P2 module hybrid configuration proposed by BorgWarner in 2017 and has been large-scale commercial use in 2019. Up to now, some HEVs models of Changan, FAW and other companies have adopted the chain coupler^10^.

The chain in HEVs coupler is a kind of rocker pin jointed silent chain which can transmit forces and velocities by the rolling movement between adjacent rocker pins, thus a higher torque can be coupled^11^. As for HEVs, the running noise of the coupler chain is directly related to the ride comfort. However, at present, there are few research on the noise influencing factors about HEVs coupler chain, and there is no effective way to reduce its running noise. Moreover, because the running noise of HEVs coupler chain is mainly derived from the flexural vibration of the chain instead of the rigid body vibration, the vibration and noise characteristics of HEVs coupler chain cannot be described by the conventional dynamic analysis^12^.

Most of the existing research on the noise of chain drive is mainly about the common chain, such as: roller chain, silent chain, leaf chain, and continuously variable transmission (CVT) chain etc., and there are few studies on the noise of HEVs coupler chain. Zhang et al.^13,14^, To reveal the noise mechanism of motorcycle chain, measured the sound pressure level (SPL) and acoustical power of the roller chain in motorcycle engine, optimized the tooth profile of the sprocket, and established an acoustic model about the dynamic response and induced sound pressure of roller. Liu et al.^15–17^, proposed a kind of split roller made of non-metallic elastic materials to reduce the noise of chain drive, and found that the noise in the silent chain transmission system can be reduced by using the silicone fluorine rubber link plate. Cheng et al.^18^, through using the simulation software, studied the noise characteristics of rounded pin jointed silent chain. Nakazawa et al.^19^, by building the vibration model for CVT chain, analyzed the influences of the different pin profile curves on the vibrations and noise performances of CVT chain. Iwai et al.^20^, proposed an additive combination method to reduce CVT chain noise by 6 dB. The above-mentioned research is helpless for analyzing the noise characteristics of HEVs coupler chain, because the vibration and noise of HEVs coupler chain is greatly affected by its special meshing, and the contact form between chain and sprocket for HEVs coupler chain is totally different from that for the common chain.

Recently, some researchers found that the polygonal action of HEVs coupler chain drive system can be decreased effectively if the inner meshing profiles can be designed for the chain plate. This is not only suitable for the general drive system, but also suitable for the multi-phase transmission system^21^. In addition, when the transmission ratio is close to 1 and the inner meshing profile is a straight line, the percentage reduce of the system polygonal action can be reached at 37%^22^. However, whether the inner meshing profile can reduce the running noise of HEVs coupler chain is controversial. Normally, the polygonal action is measured by the maximum quantities of the center distance fluctuation, its essence is the maximum amplitude of driven sprocket vibration. In conventional theory, if the polygonal action is smaller, the vibration of driven sprocket will be smaller, and the meshing will be more stable. According to the dynamics theory, the vibration of the chain should be smaller, and the running noise should be reduced^23,24^. However, Li^25^ found that the noise of the chain with the inner meshing profile is not bound to be reduced. Meng^26^ held that the noise of the chain with the inner meshing profile can be reduced or stable by adding some chain links without the inner meshing profile, thus to pursue the reduction of the chain noise is to pursue the best combination of different meshing mechanism chain links. Since no in-depth and detailed experimental analysis of the above issues has been conducted by scholars, the controversy has not been resolved. To further improve the ride comfort of HEVs with the chain coupler, the influence of the inner meshing profile on the vibration and noise of the chain should be deeply studied.

In this paper, we try to use the vibration caused by the polygonal action to evaluate the noise. Firstly, by analyzing the vibration of the pitch point of the classical HEVs coupler chain at the tension side, we analyzed the basic vibration locus and the characteristics of the basic vibration velocities and acceleration. Secondly, according to the relations under the inner meshing, we divided the meshing between the chain with the inner meshing profile and the sprockets into five processes. Based on a specific example, we proved that, under the ideal conditions, the inner meshing profile can reduce the vibration amplitude, the *Y*-axis RMS value of the vibration velocity, and the absolute maximum value of the *X*-axis/*Y*-axis vibration acceleration. Therefore, we assumed that the inner meshing profile may reduce the vibration and noise of HEVs coupler chain. Thirdly, we analyzed the influence of the error of the inner meshing profile on the chain vibration. The error will deviate the chain pitch line and disorder the meshing to instead increase the vibration amplitude, the *X*-axis vibration velocity, and the high frequency vibration. Thus, we deduced that the noise of HEVs coupler chain with the inner meshing profile might be increased at the beginning. Finally, we conducted the dynamics simulation and the noise and wear experiment to verify the correctness of the results in this paper. The results show that there is dual effect of the inner meshing profile on the noise of HEVs coupler chain, thus the controversy is solved.

## HEVs coupler chain structure

**Fig. 2:**
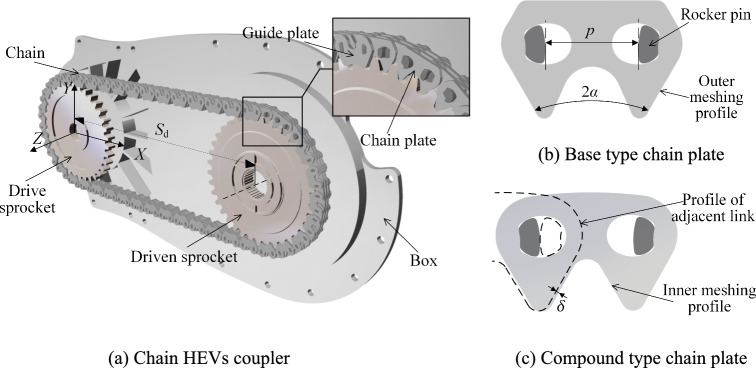
Structure of chain HEVs coupler.

It can be seen from Fig. 2a, the chain HEVs coupler is structured by a drive sprocket, a driven sprocket, a HEVs coupler chain and a box. A HEVs coupler chain is composed of several guide plates, rocker pins and chain links, and a chain link consists of several chain plates with identical projections on *XOY* plane. The distance between the drive sprocket rotation center and the driven sprocket rotation center is the system center distance *S*_d_, the tooth number for the drive sprocket and driven sprocket is *z*_1_ and *z*_2_.

For most HEVs coupler chains, the meshing between the chain and the sprockets occurs only on the outer meshing profile, and the corresponding chain plate is shown in Fig. 2b. The angle between the outer meshing profiles is the tooth profile angle 2*α*, and the minimum distance between the locating rocker pins on one chain plate is the chain pitch *p*. For convenience of illustration, the classical chain only with the outer meshing profile is called the base type HEVs coupler chain.

In this paper, we choose the straight type inner meshing profile to analyze, and there is *z*_1_ ≈ *z*_2_, as shown in Fig. 2c. The contact between the inner meshing profile and the sprocket is called the inner meshing, and the chain with the inner meshing profiles and outer meshing profiles is called the compound type HEVs coupler chain.

As for the compound type chain, the inner meshing profile extends out a distance *δ* relative to the outer meshing profile of the adjacent chain link, and *δ* should satisfy^22^:

1 $$\delta = \frac{p}{2}(\csc \frac{\pi }{z} - \cot \frac{\pi }{z})\sin \alpha$$

In addition, the compound type chain also needs to meet the following conditions^22^:

2 $$\left\{ {\begin{array}{*{20}c} {\alpha = \alpha_{11} = \alpha_{12} } \\ {p = p_{11} = p_{11} } \\ \end{array} } \right.$$

where, *p*_11_ is the drive sprocket pitch, *p*_12_ is the driven sprocket pitch, and *α*_11_ is the drive sprocket pressure angle, and *α*_12_ is the driven sprocket pressure angle.

In this paper, the parameters and its values of the HEVs coupler chain transmission system that we used are listed in Table 1.

**Table 1:** System parameters.

Symbol	Meaning	Equation	Value	Units
*p*	Chain pitch	–	9.525	mm
*α*	Half tooth profile angle	–	π/6	rad
*S*_d_	System center distance	–	228.62	mm
*z*_1_	Tooth number of drive sprocket	–	35	–
*z*_2_	Tooth number of driven sprocket	*z*_2_ > *z*_1_	37	–
*r*_p1_	Radius of the locating circle of drive sprocket	*r*_p1_ = *p*/2sin(*φ*_1_/2)^22^	53.130	mm
*r*_p2_	Radius of the locating circle of driven sprocket	*r*_p2_ = *p*/2sin(*φ*_2_/2)^22^	56.158	mm
*φ*_1_	Pitch angle of drive sprocket	*φ*_1_ = 2π/*z*_1_	0.1795	rad
*φ*_2_	Pitch angle of driven sprocket	*φ*_2_ = 2π/*z*_2_	0.1698	rad
*N*_1_	Input speed	–	500 ~ 6000	rpm
*ω*_1_	Angular velocity of drive sprocket	*ω*_1_ = *N*_1_π/30	16.67π ~ 200π	rad/s

Based on the parameter values in Table 1, there is *z*_1_ ≠ *z*_2_. According to Eq. (1), there are two possible values for *δ* in one transmission system, that are *δ*_1_ = 0.1073 mm and *δ*_2_ = 0.1015 mm. Because *δ*_1_ ≈ *δ*_2_, we select the design value of *δ* is 0.107 mm. According to Eq. (2), there are* p*_11_ = *p*_12_ = *p* = 9.525 mm and *α*_11_ = *α*_12_ = *α* = π/6.

## Basic vibration

The HEVs coupler chain is a type of flexible component, and its noise is difficult to analyze using conventional rigid body analysis methods^28^. In this paper, we mainly use the flexible vibration of the chain to evaluate the noise, assuming that the flexible vibration depends on the meshing between the chain and the sprocket, and treat the vibration of the base type chain as the basic vibration.

### Basic vibration locus

**Fig. 3:**
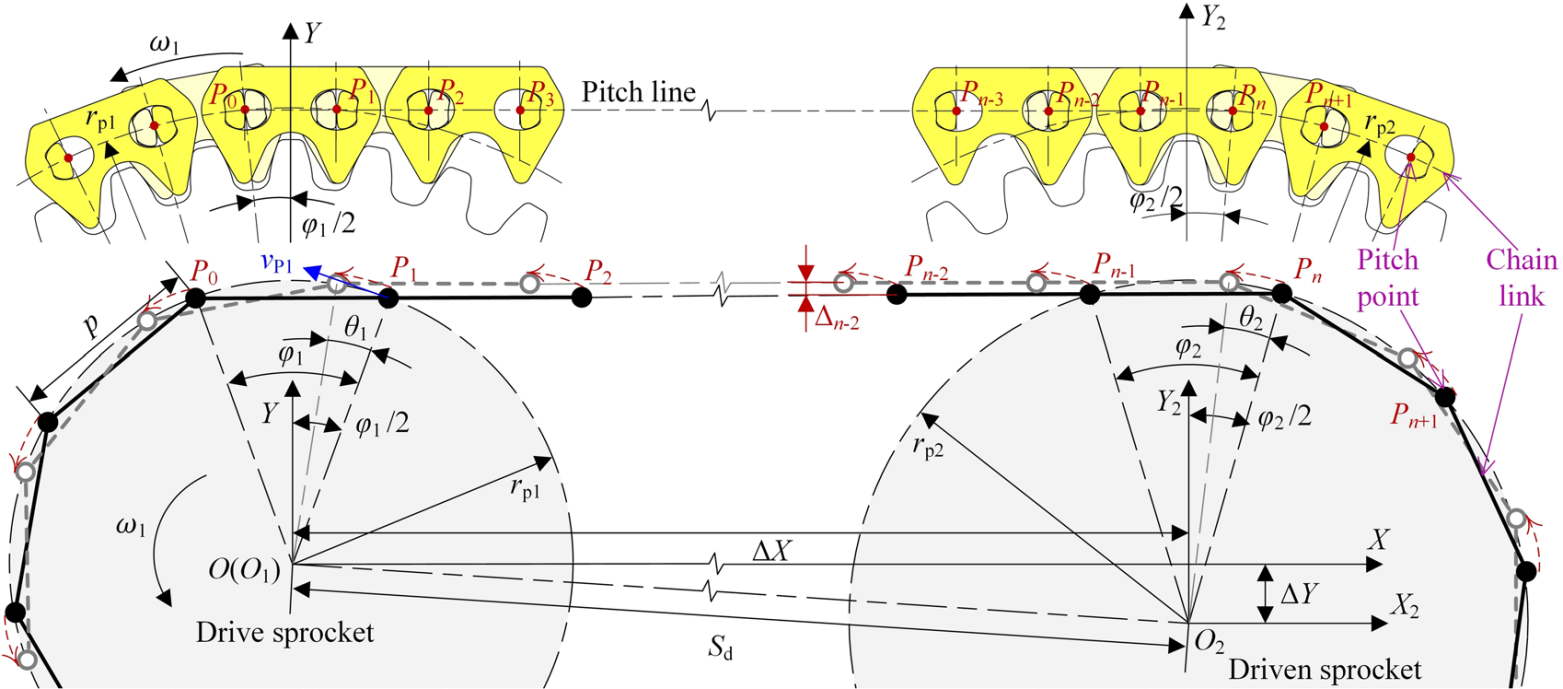
Vibration analysis model of base type HEVs coupler chain.

The pitch point of HEVs coupler chain is the intersection point between the chain pitch line and the common tangent of the rocker pins. If using the segment between adjacent pitch points to represent the chain link and using the circle which of radius is *r*_p_ to represent the sprocket, the model to analyze the vibration of the chain drive can be got, as Fig. 3 shows.

In Fig. 3, the rotation direction of the drive sprocket is anticlockwise, so the upside chain is the tension side chain that is usually chosen as the research object^18,19^. For the sake of analysis, we make the tension side chain to be horizontal. The rotation center of the drive sprocket is fixed at the origin of *S*(*XOY*), and that of the driven sprocket is fixed at the origin of *S*_2_(*X*_2_*O*_2_*Y*_2_). Supposing the distance between *X*-axis and *X*_2_-axis in the vertical direction is Δ*Y*, and the distance between *Y*-axis and *Y*_2_-axis in the horizontal direction is Δ*X*, there is:

3 $$\left\{ {\begin{array}{*{20}c} {\Delta Y = r_{{{\text{p}}2}} - r_{{{\text{p}}1}} } \\ {\Delta X = \sqrt {S_{{\text{d}}}^{2} - \Delta Y^{2} } } \\ \end{array} } \right.$$

If there are *n* pitch points on the tension side chain, the number of the free links is *n*—1. The chain pitch points at the tension side are named *P*_1_ to *P*_n_ from left to right respectively, and the coordinate of an arbitrary pitch point *P*_*i*_ is (*x*_*i*_, *y*_*i*_). The black lines and points represent the initial position of the base type chain. When ∠*P*_1_*OY* = *φ*_1_/2, the base type chain is at the initial state, chain link *P*_0_*P*_1_ is located on the drive sprocket, thus the pitch points *P*_0_ and *P*_1_ are on the locating circle of the drive sprocket. It is easily to obtain ∠*P*_*n*_*O*_2_*Y*_2_ ≈ *φ*_2_/2.

If the drive sprocket rotates *θ*_1_, the driven sprocket will rotate:

4 $$\theta_{2} = \frac{{z_{1} }}{{z_{2} }}\theta_{1}$$

The gray lines and points represent the position of the chain after the drive sprocket rotates *θ*_1_. When 0 ≤ *θ*_1_ < *φ*_1_, the highest pitch point that close to the drive sprocket is *P*_1_, and the coordinate of *P*_1_ in *S*(*XOY*) is:

5 $$\left[ {\begin{array}{*{20}c} {x_{1} } \\ {y_{1} } \\ \end{array} } \right] = \left[ {\begin{array}{*{20}c} {\sin (\frac{{\varphi_{1} }}{2} - \theta_{1} )} & 0 \\ {\cos (\frac{{\varphi_{1} }}{2} - \theta_{1} )} & 0 \\ \end{array} } \right]\left[ {\begin{array}{*{20}c} {r_{{{\text{p}}1}} } \\ 1 \\ \end{array} } \right]$$

Similarly, when 0 ≤ *θ*_2_ < *φ*_2_, the highest pitch point close to the driven sprocket is *P*_*n*_, and the coordinate of *P*_*n*_ in *S*(*XOY*) is:

6 $$\left[ {\begin{array}{*{20}c} {x_{n} } \\ {y_{n} } \\ \end{array} } \right] = \left[ {\begin{array}{*{20}c} {\sin (\frac{{\varphi_{2} }}{2} - \theta_{2} )} & {\Delta X} \\ {\cos (\frac{{\varphi_{2} }}{2} - \theta_{2} )} & { - \Delta Y} \\ \end{array} } \right]\left[ {\begin{array}{*{20}c} {r_{{{\text{p}}2}} } \\ 1 \\ \end{array} } \right]$$

Based on Eq. (5) and Eq. (6), the coordinate of *P*_*i*_ in *S*(*XOY*) is:

7 $$\left[ {\begin{array}{*{20}c} {x_{i} } \\ {y_{i} } \\ \end{array} } \right] = \left[ {\begin{array}{*{20}c} {x_{n} - x_{1} } & {x_{1} } \\ {y_{n} - y_{1} } & {y_{1} } \\ \end{array} } \right]\left[ {\begin{array}{*{20}c} {\frac{i - 1}{{n - 1}}} \\ 1 \\ \end{array} } \right]$$

Supposing the length of the tension side chain is *l*_t_, it should be:

8 $$l_{{\text{t}}} = \Delta X = (n - 1)p$$

**Fig. 4:**
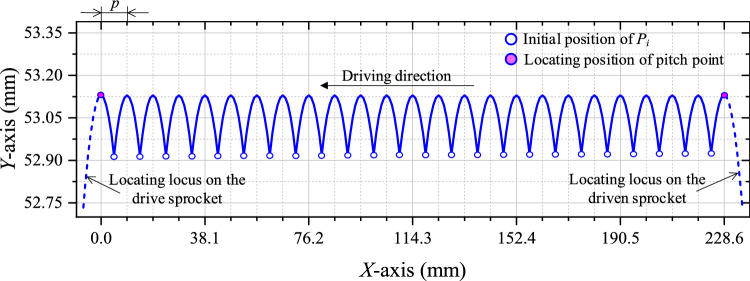
Basic vibration locus of HEVs coupler chain.

According to the values of the parameters in Table 1, combined with Eqs. (3) and (8), we can obtain *n* = 25 and *l*_t_ = 228.6 mm. According to Eqs. (4)–(7), the basic vibration locus of the chain at the tension side is shown in Fig. 4.

It can be seen from Fig. 4 that the basic vibration locus has certain sinusoidal characteristics. Supposing the vibration amplitude of *P*_*i*_ is Δ_*i*_, considering *z*_1_ is close to *z*_2_ and *z*_1_ < *z*_2_, there is:

9 $$\left\{ {\begin{array}{*{20}c} {\Delta_{1} > \Delta_{2} > \cdot \cdot \cdot > \Delta_{k} > \cdot \cdot \cdot > \Delta_{n - 1} > \Delta_{n} } \\ {\Delta_{1} \approx \Delta_{2} \approx \cdot \cdot \cdot \approx \Delta_{k} \approx \cdot \cdot \cdot \approx \Delta_{n - 1} \approx \Delta_{n} } \\ \end{array} } \right.$$

### Basic vibration velocity

As for chain drive, the vibration velocity of the tension side chain mainly depends on the actual position of the meshing-in pitch point. As Fig. 3 shows, link *P*_1_*P*_2_ is meshing with the drive sprocket, so the meshing-in pitch point is *P*_1_. Because *P*_1_ is on the locating circle of the drive sprocket, the velocity of *P*_1_ is *v*_P1_ = *ω*_1_· *r*_p1_.

Supposing time is *t*, according to the initial position of *P*_1_ in Fig. 3, the *X*-axis vibration velocity of the chain is:

10 $$v_{X} (t) = \omega_{1} r_{{{\text{p1}}}} \cdot \cos (\frac{2j + 1}{2}\varphi_{1} - \omega_{1} t), \, j\frac{{\varphi_{1} }}{{\omega_{1} }} \le t < (j + 1)\frac{{\varphi_{1} }}{{\omega_{1} }}$$

where, *j* is an arbitrary integer, it satisfies *j* ≥ 0.

Similarly, the *Y*-axis vibration velocity is:

11 $$v_{Y} (t) = \omega_{1} r_{{{\text{p1}}}} \cdot \sin (\frac{2j + 1}{2}\varphi_{1} - \omega_{1} t), \, j\frac{{\varphi_{1} }}{{\omega_{1} }} \le t < (j + 1)\frac{{\varphi_{1} }}{{\omega_{1} }}$$

According to Eqs. (10) and (11), the velocity vibration ranges in the two directions are:

12 $$\left\{ {\begin{array}{*{20}c} {v_{X\min } \sim v_{X\max } = \omega_{1} r_{{{\text{p1}}}} \cos \frac{{\varphi_{1} }}{2}\sim \omega_{1} r_{{{\text{p1}}}} } \\ {v_{Y\min } \sim v_{Y\max } = - \omega_{1} r_{{{\text{p1}}}} \sin \frac{{\varphi_{1} }}{2}\sim \omega_{1} r_{{{\text{p1}}}} \sin \frac{{\varphi_{1} }}{2}} \\ \end{array} } \right.$$

Normally, the degree of the simple harmonic vibration in the medium frequency range is evaluated by the root mean square(RMS) value of the vibration velocity. Moreover, the RMS value is greater, the medium frequency vibration or noise is greater^27^. The RMS value of *v*_*X*_ is:

13 $$v_{{X{\text{R}}}} = \sqrt {\frac{{\int_{0}^{T} {[\omega_{1} r_{{{\text{p1}}}} \cdot \cos (\frac{{\varphi_{1} }}{2} - \omega_{1} t)]^{2} {\text{d}}t} }}{T}} = \omega_{1} r_{{{\text{p1}}}} \sqrt {\frac{{\varphi_{1} + \sin \varphi_{1} }}{{2\varphi_{1} }}}$$

The RMS value of *v*_*Y*_ is:

14 $$v_{{Y{\text{R}}}} = \sqrt {\frac{{\int_{0}^{T} {[\omega_{1} r_{{{\text{p1}}}} \cdot \sin (\frac{{\varphi_{1} }}{2} - \omega_{1} t)]^{2} {\text{d}}t} }}{T}} = \omega_{1} r_{{{\text{p1}}}} \sqrt {\frac{{\varphi_{1} - \sin \varphi_{1} }}{{2\varphi_{1} }}}$$

It can be known from Eq. (12) that the *X*-axis velocity vibration is not belong to a simple harmonic vibration. Therefore, we try to use the relative RMS value to evaluate the *X*-axis medium frequency vibration of the chain and the formula is:

15 $$v_{{X{\text{R0}}}} = v_{{X{\text{R}}}} - v_{X\min }$$

**Fig. 5:**
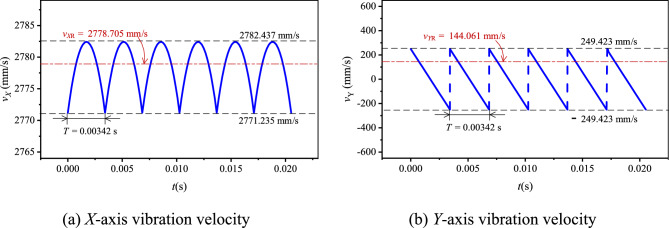
Basic vibration velocity curves of HEVs coupler chain.

Let the input speed *N*_1_ be 500 rpm(*ω*_1_ = 16.67π rad/s). Based on the values in Table 1, combined with Eq. (12), there are *v*_*X*_ = 2771.235–2782.437 mm/s, *v*_*Y*_ = − 249.423–249.423 mm/s. According to Eqs. (13) and (14), there are *v*_*X*R_ = 2778.705 mm/s, *v*_*Y*R_ = 144.061 mm/s. Based on Eq. (15), we can obtain *v*_*X*R0_ = 2778.705–2771.235 = 7.470 mm/s. According to Eqs. (10) and (11), the basic vibration velocity curves of HEVs coupler chain are shown in Fig. 5.

### Basic vibration acceleration

The acceleration function of the chain in the horizontal direction is the derivative of Eq. (10):

16 $$a_{X} (t) = \frac{{{\text{d}}v_{X} (t)}}{{{\text{d}}t}} = r_{{{\text{p1}}}} \omega_{1}^{2} \sin (\frac{2j + 1}{2}\varphi_{1} - \omega_{1} t), \, j\frac{{\varphi_{1} }}{{\omega_{1} }} \le t < (j + 1)\frac{{\varphi_{1} }}{{\omega_{1} }}$$

Similarly, based on Eq. (11), there is:

17 $$a_{Y} (t) = \frac{{{\text{d}}v_{Y} (t)}}{{{\text{d}}t}} = - r_{{{\text{p1}}}} \omega_{1}^{2} \cos (\frac{2j + 1}{2}\varphi_{1} - \omega_{1} t), \, j\frac{{\varphi_{1} }}{{\omega_{1} }} \le t < (j + 1)\frac{{\varphi_{1} }}{{\omega_{1} }}$$

Thus, the acceleration vibration ranges in the horizontal and vertical directions are:

18 $$\left\{ {\begin{array}{*{20}c} {a_{X\min } \sim a_{X\max } = - r_{{{\text{p1}}}} \omega_{1}^{2} \sin \frac{{\varphi_{1} }}{2}\sim r_{{{\text{p1}}}} \omega_{1}^{2} \sin \frac{{\varphi_{1} }}{2}} \\ {a_{Y\min } \sim a_{Y\max } = - r_{{{\text{p1}}}} \omega_{1}^{2} \sim - r_{{{\text{p1}}}} \omega_{1}^{2} \cos \frac{{\varphi_{1} }}{2}} \\ \end{array} } \right.$$

According to the vibration and noise theory, the maximum absolute value of the acceleration can be used to measure the vibration and noise in the high frequency range. Commonly, the maximum absolute value is greater, the high frequency vibration or noise is greater^27^. In this study, there is:

19 $$\left[ {\begin{array}{*{20}c} {|a_{X} |_{\max } } \\ {|a_{Y} |_{\max } } \\ \end{array} } \right] = \left[ {\begin{array}{*{20}c} {|a_{X} (t)|_{\max } } \\ {|a_{Y} (t)|_{\max } } \\ \end{array} } \right] = \left[ {\begin{array}{*{20}c} {a_{X} (t)_{\max } } \\ { - a_{Y} (t)_{\min } } \\ \end{array} } \right] = \left[ {\begin{array}{*{20}c} {r_{{{\text{p}}1}} \omega_{1}^{2} \sin \frac{{\varphi_{1} }}{2}} \\ {r_{{{\text{p}}1}} \omega_{1}^{2} } \\ \end{array} } \right]$$

**Fig. 6:**
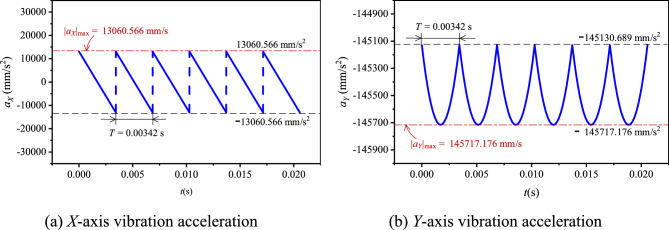
Basic vibration acceleration curves of HEVs coupler chain.

When *N*_1_ = 500 rpm, according to the values in Table 1, combined with Eqs. (18) and (19), we can obtain *a*_*X*_ = − 13,060.566–13,060.566 mm/s, *a*_*Y*_ = −145,717.176 to − 145,130.689 mm/s^2^, |*a*_*X*_|_max_ = 13,060.566 mm/s^2^, and |*a*_*Y*_|_max_ = 145,717.176 mm/s^2^. Based on Eqs. (16) and (17), the basic vibration acceleration curves of the chain in the two directions can be got, as shown in Fig. 6.

## Inner meshing profile influences

In previous research, there has been little discussion on the influence of the inner meshing profile on the vibration and noise of HEVs coupler chain. Due to the lack of understanding of the noise generation mechanisms, the application of the compound type chain has been significantly restricted.

### Influence on vibration locus

**Fig. 7:**
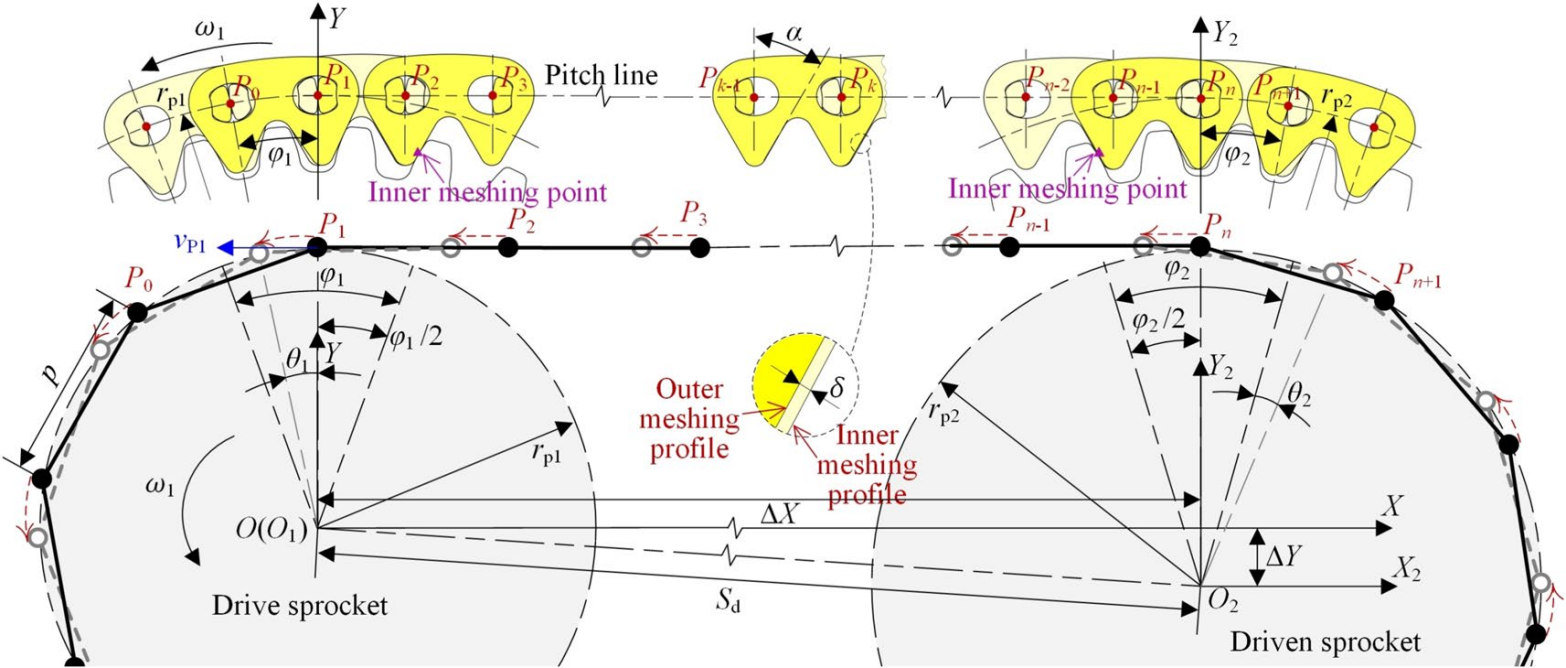
Vibration analysis model of compound type HEVs coupler chain.

Figure 7 shows the vibration analysis model of the compound type HEVs coupler chain. According to the involute meshing theory^26^, under the effect of *δ*, link *P*_2_*P*_3_ and link *P*_*n*−2_*P*_*n*−1_ will continuously mesh with the drive sprocket and the driven sprocket, so that the *Y*-axis coordinates of *P*_2_ and *P*_*n*−1_ will be not changed. The contact point between the chain and the sprockets is called the inner meshing point. Obviously, if this inner meshing exists throughout the whole meshing cycle, the vibration of the tension side chain in the vertical direction will be zero.

Obviously, the inner meshing cannot exist throughout the meshing cycle. This is because the chain plate hole of HEVs coupler chain is obviously greater than that of the normal chain. To increase the strength of the chain plate, the tooth shape of the sprocket can only be reduced, so the inner meshing zone in the sprocket tooth is incomplete^22^. Therefore, the vibration locus of HEVs coupler chain with the inner meshing profile should be further discussed.

To facilitate the analysis, we suppose that the chain system is in its initial state when pitch point *P*_1_ is on the *Y*-axis, as Fig. 7 shows. According to the meshing form between the chain and the sprockets, the meshing of the compound type HEVs coupler chain can be divided into five processes. When the rotation angle of the drive sprocket is equal to *η*_1_, *η*_2_, *η*_3_, and *η*_4_ respectively (*η*_1_ < *η*_2_ < *η*_3_ < *η*_4_), the meshing form will be changed.

#### The first process

In the first process, the rotation angle of the driven sprocket satisfies 0 ≤ *θ*_2_ < *η*_21_, the rotation angle of the drive sprocket satisfies 0 ≤ *θ*_1_ < *η*_1_ (*η*_1_ = *η*_21_ ·*z*_2_/*z*_1_), and the meshing form between the tension side chain and the sprockets are both inner meshing. As Fig. 7 shows, the right side inner meshing profile of link *P*_*n*−2_*P*_*n*−1_ is meshing with the driven sprocket tooth, and the left side inner meshing profile of link *P*_2_*P*_3_ is meshing with the drive sprocket tooth. According to the involute meshing theory^26^, the pitch line of the tension side chain is horizontal, and it is a common tangent line of the sprockets. We treat the position of the tension side chain pitch line in this process as the ideal position. The coordinate of pitch point *P*_*i*_ (1 < *i* < *n*) should satisfy:

20 $$\left[ {\begin{array}{*{20}c} {x_{i} } \\ {y_{i} } \\ \end{array} } \right] = \left[ {\begin{array}{*{20}c} {i - 1} & { - \theta_{1} } \\ 0 & 1 \\ \end{array} } \right]\left[ {\begin{array}{*{20}c} p \\ {r_{{{\text{p1}}}} } \\ \end{array} } \right]$$

At this time, because *P*_1_ is locating on the drive sprocket, its coordinate should satisfy Eq. (5).

**Fig. 8:**
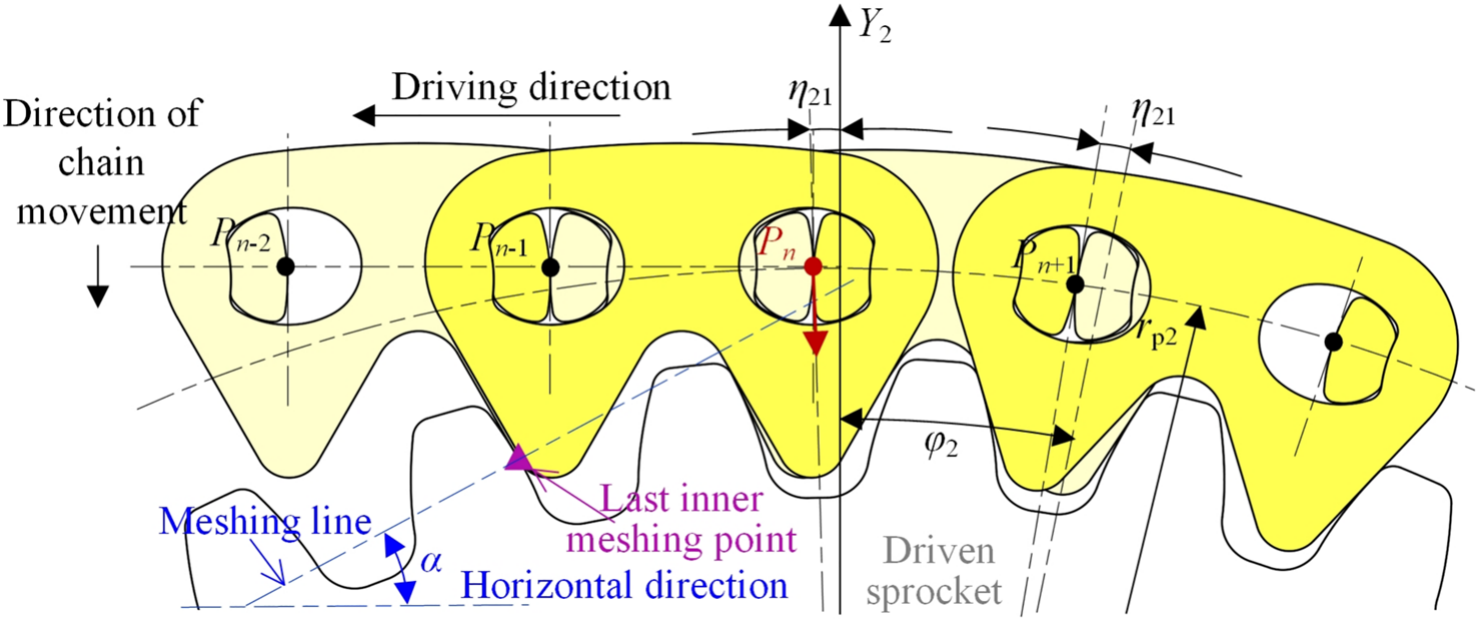
Imminent separation between the chain inner meshing profile and the driven sprocket.

When *θ*_2_ = *η*_21_, the right side inner meshing profile of link *P*_*n*−2_*P*_*n*−1_ begins to separate from the driven sprocket tooth. As Fig. 8 displays, *P*_n_ will move in the radial direction from the ideal position of the pitch line to the locating circle of the driven sprocket, so that link *P*_*n*_*P*_*n*+1_ is outer meshing with the driven sprocket. The meshing from Figs. 7 to 8 is called the first process in this paper.

#### The second process

In the second process, there are *η*_21_ ≤ *θ*_2_ < *η*_22_ and *η*_1_ ≤ *θ*_1_ < *η*_2_ (*η*_1_ = *η*_21_ ·*z*_2_/*z*_1_), link *P*_*n*_*P*_*n*+1_ is locating on the driven sprocket, and link *P*_2_*P*_3_ is inner meshing with the drive sprocket.

The highest pitch point that close to the drive sprocket is *P*_3_, the coordinate is:

21 $$\left[ {\begin{array}{*{20}c} {x_{3} } \\ {y_{3} } \\ \end{array} } \right] = \left[ {\begin{array}{*{20}c} {4\sin \frac{{\varphi_{1} }}{2} - \theta_{1} } & 0 \\ 1 & 0 \\ \end{array} } \right]\left[ {\begin{array}{*{20}c} {r_{{{\text{p1}}}} } \\ 1 \\ \end{array} } \right]$$

The highest pitch point that close to the driven sprocket is *P*_*n*_, the coordinate is:

22 $$\left[ {\begin{array}{*{20}c} {x_{n} } \\ {y_{n} } \\ \end{array} } \right] = \left[ {\begin{array}{*{20}c} { - \sin \theta_{2} } & {\Delta X} \\ {\cos \theta_{2} } & { - \Delta Y} \\ \end{array} } \right]\left[ {\begin{array}{*{20}c} {r_{{{\text{p2}}}} } \\ 1 \\ \end{array} } \right]$$

According to Eqs. (21) and (22), we can obtain the coordinates of *P*_*i*_(3 < *i* < *n*).

23 $$\left[ {\begin{array}{*{20}c} {x_{i} } \\ {y_{i} } \\ \end{array} } \right] = \left[ {\begin{array}{*{20}c} {x_{n} - x_{3} } & {x_{3} } \\ {y_{n} - y_{3} } & {y_{3} } \\ \end{array} } \right]\left[ {\begin{array}{*{20}c} {\frac{i - 3}{{n - 3}}} \\ 1 \\ \end{array} } \right]$$

**Fig. 9:**
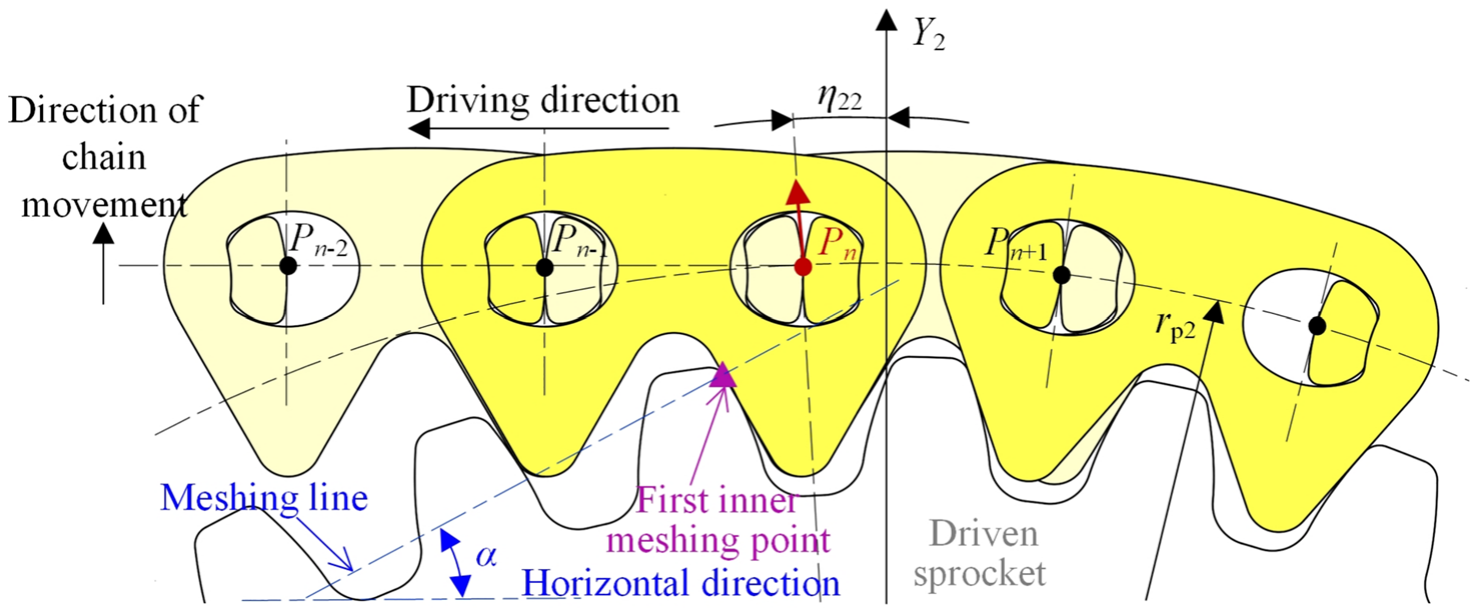
Imminent contact between the chain inner meshing profile and the driven sprocket.

As shown in Fig. 9, when the rotation angle of the driven sprocket is *θ*_2_ = *η*_22_, the right side inner meshing profile of link *P*_*n*−1_*P*_*n*_ will begin to contact with the driven sprocket, *P*_n_ will move in the radial direction from the location position to the ideal position of the pitch line, and the meshing form of the tension side chain and sprockets will be changed back to the inner meshing. The meshing process from Figs. 8 to 9 is called the second process.

#### The third process

In the third process, there is *η*_2_ ≤ *θ*_1_ < *η*_3_. During the third process, the tension side chain is inner meshing with the drive sprocket and the driven sprocket, so the coordinates of *P*_*i*_ satisfy Eq. (20).

**Fig. 10:**
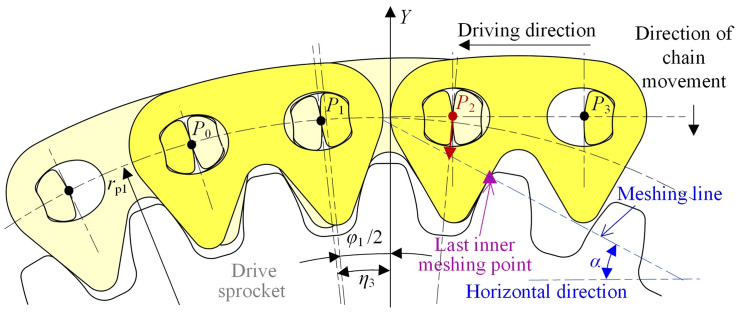
Imminent separation between the chain inner meshing profile and the drive sprocket.

When *θ*_1_ = *η*_3_, the left side inner meshing profile of link *P*_2_*P*_3_ will begin to separate from the drive sprocket tooth, so that *P*_2_ will move in the radial direction from the ideal position to the locating circle of the drive sprocket, to make link *P*_1_*P*_2_ locating on the drive sprocket, as Fig. 10 illustrates. The meshing process from Figs. 9 to 10 is the third process.

#### The fourth process

In the fourth process, there is *η*_3_ ≤ *θ*_1_ < *η*_4_, the meshing form for the drive sprocket is the outer meshing, and that for the driven sprocket is the inner meshing.

The highest pitch point that close to the drive sprocket is *P*_2_, and the coordinate is:

24 $$\left[ {\begin{array}{*{20}c} {x_{2} } \\ {y_{2} } \\ \end{array} } \right] = \left[ {\begin{array}{*{20}c} {\sin (\varphi_{1} - \theta_{1} )} & 0 \\ {\cos (\varphi_{1} - \theta_{1} )} & 0 \\ \end{array} } \right]\left[ {\begin{array}{*{20}c} {r_{{{\text{p1}}}} } \\ 1 \\ \end{array} } \right]$$

The highest pitch point that close to the driven sprocket is *P*_*n*−1_, and there is:

25 $$\left[ {\begin{array}{*{20}c} {x_{n - 1} } \\ {y_{n - 1} } \\ \end{array} } \right] = \left[ {\begin{array}{*{20}c} {(n - 1)} & { - \theta_{1} } \\ 0 & 1 \\ \end{array} } \right]\left[ {\begin{array}{*{20}c} p \\ {r_{{{\text{p1}}}} } \\ \end{array} } \right]$$

Based on Eqs. (24) and (25), we can obtain the coordinates of *P*_*i*_(2 < *i* < *n*), that are:

26 $$\left[ {\begin{array}{*{20}c} {x_{i} } \\ {y_{i} } \\ \end{array} } \right] = \left[ {\begin{array}{*{20}c} {x_{n - 1} - x_{2} } & {x_{2} } \\ {y_{n - 1} - y_{2} } & {y_{2} } \\ \end{array} } \right]\left[ {\begin{array}{*{20}c} {\frac{i - 2}{{n - 3}}} \\ 1 \\ \end{array} } \right]$$

**Fig. 11:**
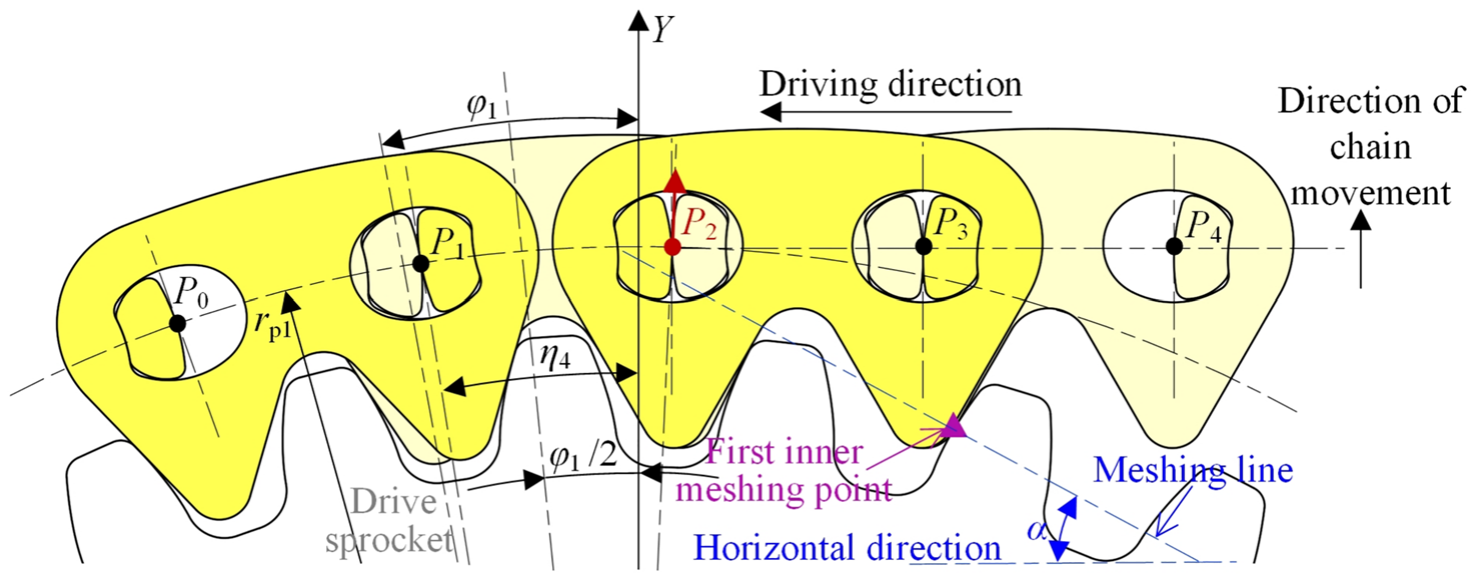
Imminent contact between the chain inner meshing profile and the drive sprocket.

Figure 11 shows the relations between the inner meshing profile of the tension side chain and the drive sprocket when *θ*_1_ = *η*_4_. The left side inner meshing profile of link *P*_3_*P*_4_ will begin to separate from the drive sprocket tooth, so that the tension side chain will both contact with the drive sprocket and the driven sprocket by the inner meshing. The meshing process from Figs. 10 to 11 is called the fourth process.

#### The fifth process

**Fig. 12:**
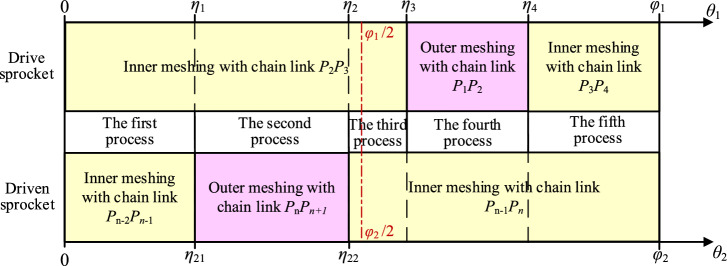
Meshing forms under different rotation angles.

The meshing process from Fig. 11 back to Fig. 7is referred as the fifth process. During the fifth process, the tension side chain will both contact with the drive sprocket and the driven sprocket by the inner meshing, and the coordinate of *P*_*i*_ satisfies Eq. (20). According to Figs. 7, 8, 9, 10 and 11, the relations among the meshing forms and the sprocket rotation angles can be summarized as Fig. 12. By measuring, the values of *η*_1_, *η*_2_, *η*_3_, and *η*_4_ in this paper are 0.031 rad, 0.086 rad, 0.094 rad, and 0.148 rad respectively.

**Fig. 13:**
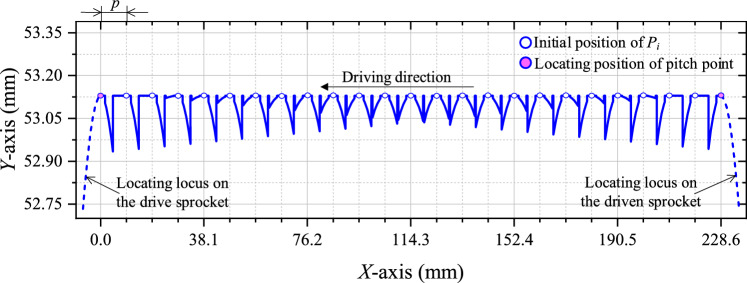
Vibration locus of HEV coupler chain with inner meshing profile.

Based on the parameter values in Table 1 and Eqs. (20)–(26), the vibration locus of the compound type HEVs coupler chain at the tension side can be calculated, as shown in Fig. 13.

Supposing the vibration amplitude of *P*_*i*_ for the compound type chain is Δ^′^_*i*_, it can be obtained from Fig. 13 that:

27 $$\Delta ^{\prime}_{{1}} > \Delta ^{\prime}_{2} > \cdot \cdot \cdot > \Delta ^{\prime}_{{\frac{(1 + n)}{2}}} < \cdot \cdot \cdot < \Delta ^{\prime}_{n - 1} < \Delta ^{\prime}_{n}$$

**Fig. 14:**
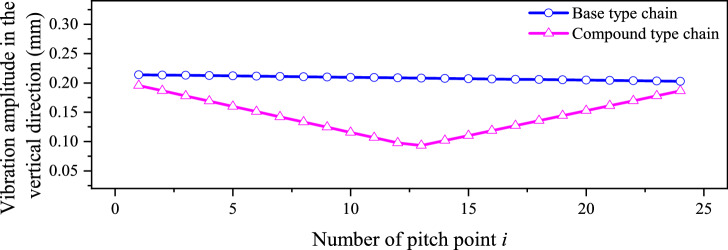
Comparison about vibration amplitude.

According to the vibration locus in Figs. 4 and 13, we can compare the vibration amplitude of the base type chain and that of the compound type chain, as Fig. 14 demonstrates.

It can be seen from Fig. 14 that, no matter what the value of *i* is, there is Δ_*i*_^′^ < Δ_*i*_. By using the inner meshing profile, the vibration amplitude of HEVs coupler chain will be reduced by:

28 $$c_{{{\text{amp}}}} = \sum\limits_{i = 1}^{n} {\frac{{\Delta_{i} - \Delta ^{\prime}_{i} }}{{\Delta_{i} }}} \times 100\%$$

According to the values in Fig. 14, we can obtain *c*_amp_ = 31.25%. Because the vibration amplitude mainly affects the noise in the low frequency range^27^, the low frequency noise of HEVs coupler chain can be effectively reduced by the inner meshing profile.

### Influence on vibration velocity

As Fig. 7 shows, when the tension side chain is inner meshing with the drive sprocket, the vibration velocity of the chain can be roughly regarded as:

29 $$\left[ {\begin{array}{*{20}c} {v_{X} } \\ {v_{Y} } \\ \end{array} } \right] = \left[ {\begin{array}{*{20}c} {\omega_{1} r_{{{\text{p1}}}} } \\ 0 \\ \end{array} } \right]$$

When the tension side chain is outer meshing with the drive sprocket, the meshing-in pitch point is on the locating circle of the drive sprocket, the vibration velocities in the horizontal and vertical directions are similar to Eqs. (10) and (11).

Based on Fig. 12, Eqs. (10) and (29), the *X*-axis vibration velocity of the tension side chain is:

30 $$v_{X} (t) = \left\{ {\begin{array}{*{20}l} {\omega_{1} r_{{{\text{p1}}}} \cdot \cos (\varphi_{1} - \omega_{1} t), \, \frac{{\eta_{3} }}{{\omega_{1} }} + j\frac{{\varphi_{1} }}{{\omega_{1} }} \le t < \frac{{\eta_{4} }}{{\omega_{1} }}{ + }j\frac{{\varphi_{1} }}{{\omega_{1} }} \, } \hfill \\ {\omega_{1} r_{{{\text{p1}}}} ,\quad {\text{others}}} \hfill \\ \end{array} } \right.$$

Similarly, *Y*-axis vibration velocity is:

31 $$v_{Y} (t) = \left\{ {\begin{array}{*{20}l} {\omega_{1} r_{{{\text{p1}}}} \cdot \sin (\varphi_{1} - \omega_{1} t), \, \frac{{\eta_{3} }}{{\omega_{1} }} + j\frac{{\varphi_{1} }}{{\omega_{1} }} \le t < \frac{{\eta_{4} }}{{\omega_{1} }}{ + }j\frac{{\varphi_{1} }}{{\omega_{1} }}} \hfill \\ {0,\quad {\text{others}}} \hfill \\ \end{array} } \right.$$

Based on Eq. (30) and Eq. (31), there are:

32 $$\left\{ {\begin{array}{*{20}l} {v^{\prime}_{X\min } \sim v^{\prime}_{X\max } = r_{{{\text{p}}1}} \omega_{1} \cos (\varphi_{1} - \eta_{3} )\sim r_{{{\text{p}}1}} \omega_{1} } \hfill \\ {v^{\prime}_{Y\min } \sim v^{\prime}_{Y\max } = 0\sim r_{{{\text{p1}}}} \omega_{1} \sin (\varphi_{1} - \eta_{3} )} \hfill \\ \end{array} } \right.$$

The RMS value of *v*_*X*_ for the compound type chain is:

33 $$\begin{aligned} v^{\prime}_{{X{\text{R}}}} & = \sqrt {\frac{{\int_{0}^{{\frac{{\eta_{3} }}{{\omega_{1} }}}} {(\omega_{1} r_{{{\text{p1}}}} )^{2} {\text{d}}t + \int_{{\frac{{\eta_{4} }}{{\omega_{1} }}}}^{T} {(\omega_{1} r_{{{\text{p1}}}} )^{2} {\text{d}}t} } + \int_{{\frac{{\eta_{3} }}{{\omega_{1} }}}}^{{\frac{{\eta_{4} }}{{\omega_{1} }}}} {[\omega_{1} r_{{{\text{p1}}}} \cdot \cos (\varphi_{1} - \omega_{1} t)]^{2} {\text{d}}t} }}{T}} \\ & = \omega_{1} r_{{{\text{p1}}}} \sqrt {1 + \frac{{2\eta_{3} - 2\eta_{4} + \sin (2\varphi_{1} - 2\eta_{3} ) - \sin (2\varphi_{1} - 2\eta_{4} )}}{{4\varphi_{1} }}} \\ \end{aligned}$$

The RMS value of *v*_*Y*_ for the compound type chain should satisfy:

34 $$v^{\prime}_{{Y{\text{R}}}} = \sqrt {\frac{{\int_{{\frac{{\eta_{3} }}{{\omega_{1} }}}}^{{\frac{{\eta_{4} }}{{\omega_{1} }}}} {[\omega_{1} r_{{{\text{p1}}}} \cdot \sin (\varphi_{1} - \omega_{1} t)]^{2} {\text{d}}t} }}{T}} = \frac{{\omega_{1} r_{{{\text{p1}}}} }}{2}\sqrt {\frac{{2\eta_{4} - 2\eta_{3} + \sin (2\varphi_{1} - 2\eta_{4} ) - \sin (2\varphi_{1} - 2\eta_{3} )}}{{\varphi_{1} }}}$$

**Fig. 15:**
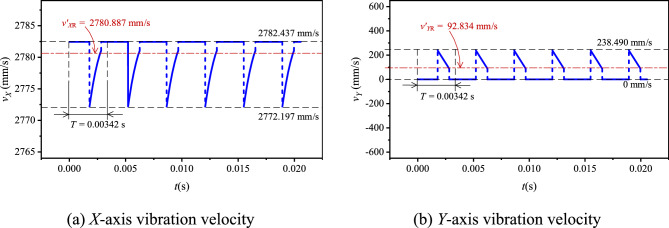
Vibration velocity curves of HEV coupler chain with inner meshing profile.

When *N*_1_ = 500 rpm (*ω*_1_ = 16.67π rad/s), according to the values in Table 1 and Eqs. (30)–(34), we can obtain the relative RMS value of *v*_*X*_ is 2780.887–2772.197 = 8.690 mm/s, and the vibration velocity curves for the compound type HEVs coupler chain in the two directions, as Fig. 15 demonstrates.

Compared with the basic vibration velocity, the percentage reduction of the relative RMS value of *v*_*X*_ for the compound type chain can be calculated by:

35 $$c_{vX} = \frac{{(v_{{X{\text{R}}0}} - v^{\prime}_{{X{\text{R}}0}} )}}{{v_{{X{\text{R}}0}} }} \times 100\%$$

The percentage reduction of the RMS value of *v*_*Y*_ for the compound type chain is:

36 $$c_{vY} = \frac{{(v_{{Y{\text{R}}}} - v^{\prime}_{{Y{\text{R}}}} )}}{{v_{{Y{\text{R}}}} }} \times 100\%$$

**Fig. 16:**
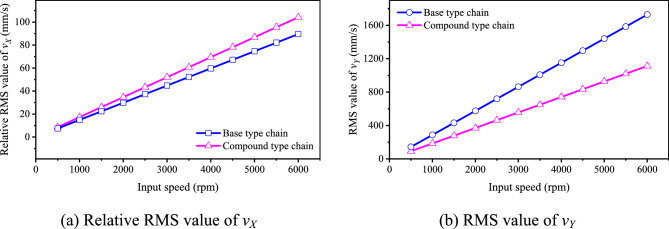
RMS values of velocities under different input speeds.

According to Eqs. (35) and (36), we can obtain *c*_*vX*_ = − 16.34% and *c*_*vY*_ = 35.56%. It means that, when *N*_1_ = 500 rpm, the inner meshing profile may increase the medium frequency vibration in the horizontal direction but reduce that in the vertical direction. To further analyze the influence of the inner meshing profile on the vibration velocity at the other speeds, we calculate the values of *v*_*X*R0_(or *v*_*X*R0_^′^) and *v*_*Y*R_(or *v*_*Y*R_^′^) under different input speeds, the results are shown in Fig. 16.

Based on Fig. 16, we can know that, whether the base type chain or the compound type chain, the RMS values of velocities will increase with the increase of the input speed, and shows a certain linearity. Therefore, the values of *c*_*vX*_ and *c*_*vY*_ will not change with the input speed.

As for the chain drive, the medium frequency noise mainly comes from the *Y*-axis velocity vibration of the chain, and the *X*-axis velocity vibration mainly causes the velocity fluctuation of the driven sprocket^18^. Considering there is |*c*_*vX*_|< *c*_*vY*_, we hold that, under the influence of the inner meshing profile, the medium frequency noise of HEVs coupler chain will be reduced.

### Influence on vibration acceleration

By derivation of Eq. (30), the *X*-axis vibration acceleration of the chain is:

37 $$a_{X} (t) = \frac{{{\text{d}}v_{X} (t)}}{{{\text{d}}t}} = \left\{ {\begin{array}{*{20}l} {r_{{{\text{p1}}}} \omega_{1}^{2} \sin (\varphi_{1} - \omega_{1} t),\frac{{\eta_{3} }}{{\omega_{1} }} + j\frac{{\varphi_{1} }}{{\omega_{1} }} \le t < \frac{{\eta_{4} }}{{\omega_{1} }}{ + }j\frac{{\varphi_{1} }}{{\omega_{1} }}} \hfill \\ {0,\quad {\text{others}}} \hfill \\ \end{array} } \right.$$

Similarly, based on Eq. (31), we can obtain:

38 $$a_{Y} (t) = \frac{{{\text{d}}v_{Y} (t)}}{{{\text{d}}t}} = \left\{ {\begin{array}{*{20}l} { - r_{{{\text{p1}}}} \omega_{1}^{2} \cos (\varphi_{1} - \omega_{1} t), \, \frac{{\eta_{3} }}{{\omega_{1} }} + j\frac{{\varphi_{1} }}{{\omega_{1} }} \le t < \frac{{\eta_{4} }}{{\omega_{1} }}{ + }j\frac{{\varphi_{1} }}{{\omega_{1} }} \, } \hfill \\ {0,\quad {\text{others}}} \hfill \\ \end{array} } \right.$$

Supposing the absolute maximum accelerations of the compound type HEVs coupler chain in the two directions are |*a*_*X*_^′^|_max_ and |*a*_*Y*_^′^|_max_, there are:

39 $$\left[ {\begin{array}{*{20}c} {|a^{\prime}_{X} |_{\max } } \\ {|a^{\prime}_{Y} |_{\max } } \\ \end{array} } \right] = \left[ {\begin{array}{*{20}c} {r_{{{\text{p1}}}} \omega_{1}^{2} \sin (\varphi_{1} - \eta_{3} )} \\ {r_{{{\text{p1}}}} \omega_{1}^{2} \cos (\varphi_{1} - \eta_{4} )} \\ \end{array} } \right]$$

**Fig. 17:**
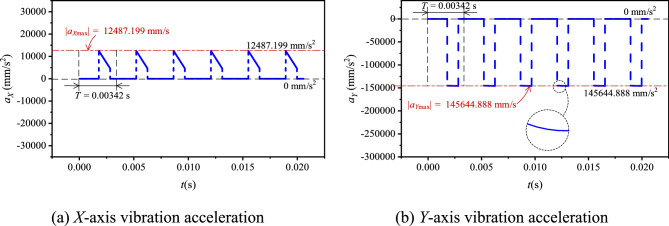
Vibration acceleration curves of HEVs coupler chain with inner meshing profile.

When *N*_1_ = 500 rpm, according to Eq. (37) ~ Eq. (39), the vibration acceleration curves of the compound type chain can be got, as shown in Fig. 17.

Compared with the base type chain, the percentage reduction of the absolute maximum *X*-axis vibration acceleration of the compound type chain can be calculated by:

40 $$c_{aX} = \frac{{(|a_{X} |_{\max } - |a^{\prime}_{X} |_{\max } )}}{{|a_{X} |_{\max } }} \times 100\%$$

Similarly, there is:

41 $$c_{aY} = \frac{{(|a_{Y} |_{\max } - |a^{\prime}_{Y} |_{\max } )}}{{|a_{Y} |_{\max } }} \times 100\%$$

**Fig. 18:**
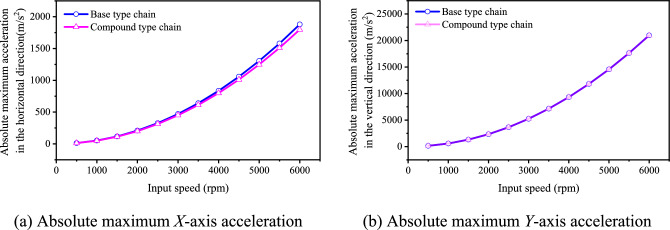
Absolute maximum acceleration under different input speeds.

Combining with the results in “Basic vibration acceleration” section, there are *c*_*aX*_ = 4.39% and *c*_*aY*_ = 0.05%. To further analyze, we also calculate the absolute maximum acceleration at different input speeds in the two directions for the two types of chain, and the results are displayed in Fig. 18. It can be seen from the figure that, at different input speeds, there are |*a*_*X*_^′^|_max_ <|*a*_*X*_|_max_ and |*a*_*Y*_^′^|_max_ ≈ |*a*_*Y*_|_max_. From the perspective of absolute maximum acceleration, the inner meshing profile can lightly reduce the vibration and noise in the high frequency range.

Moreover, the absolute maximum acceleration of chain point at two directions also determine the wear between adjacent rocker pins^11^. If the maximum acceleration is greater, the degree of the wear is greater. Therefore, we can also assume that, with the time going on, the increase of the noise for the compound type chain will be smaller than that for the base type chain.

## Error influence

In traditional research on silent chains, errors are often considered to have no effect on the meshing. However, we argue that the error of the inner meshing profile not only affects the meshing performance but also influences the drive noise of the chain.

### Error analysis

The effect of the manufacturing error of the outer meshing profile on the chain drive can be ignored. This is because the outer meshing is a kind of locating and the preloading process is based on the proper locating between the chain and the sprocket, the influence can be reduced as much as possible after preloading process^26^.

However, the error of the inner meshing profile cannot be ignored. This is because the value of *δ* cannot be manufactured precisely, and the design error and the manufacturing error will exist at the same time. For example, in this paper, we select the value of *δ* is 0.107 mm. Because the chain plate is manufactured by stamping process which cannot guarantee the accuracy of 0.001 mm, we can only take the design value of *δ* as 0.11 mm. Thus, *δ* will have a design error that is equal to 0.003 mm. Under the effect of the stamping process and preloading process, the actual error will be much bigger than 0.01 mm. Moreover, the actual error is not only difficult to control, but also difficult to measure.

**Fig. 19:**
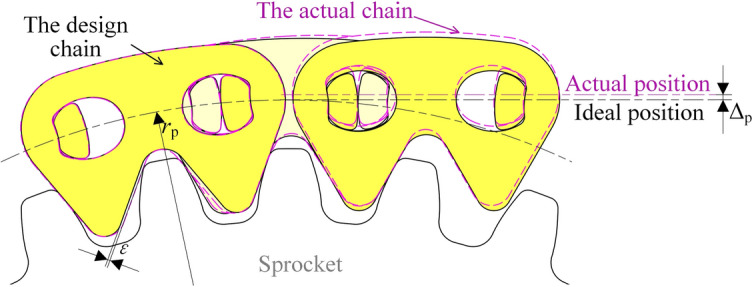
Influence of inner meshing profile error on pitch line.

As Fig. 19 shows, if the extension of the ideal inner meshing profile is *δ*, and that of the actual inner meshing profile is *δ*^′^, the final error will be:

42 $$\varepsilon = \delta ^{\prime} - \delta$$

Under the effect of *ε*, the chain pitch line will deviate from its ideal position in the vertical direction. Based on the geometrical relations in Fig. 19, the deviation satisfies:

43 $$\Delta_{{\text{p}}} = \frac{\varepsilon }{\sin \alpha }$$

In this paper, *α* = π/6, so there is Δ_p_ = 2*ε*.

### Influence of pitch line deviation

Supposing the actual vibration amplitude of the compound type HEVs coupler chain is Δ_*i*-er_, according to the inner meshing relations in Figs. 7, 13, and 19, we can obtain:

44 $$\Delta ^{\prime}_{{i - {\text{er}}}} = \Delta ^{\prime}_{i} + \Delta_{{\text{p}}}$$

Under the effect of the inner meshing profile error, Eq. (28) will be changed to:

45 $$c_{{{\text{amp}}}} = \sum\limits_{i = 1}^{n} {\frac{{\Delta_{i} - \Delta ^{\prime}_{{i - {\text{er}}}} }}{{\Delta_{i} }}} \times 100\% = \sum\limits_{i = 1}^{n} {\frac{{\Delta_{i} - \Delta ^{\prime}_{i} - \Delta_{{\text{p}}} }}{{\Delta_{i} }}} \times 100\%$$

Comparing Eqs. (45) and (28), it is no doubt that the value of *c*_amp_ is reduced.

**Fig. 20:**
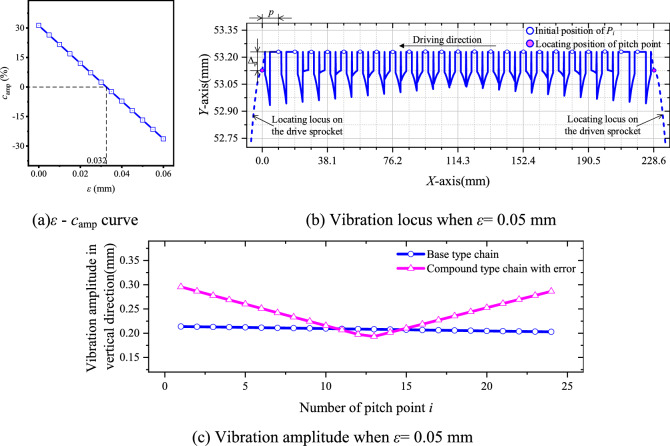
Influence of *ε* on vibration locus of HEVs coupler chain.

Based on Eq. (45) and the values in Table 1, we can obtain the relation between *ε* and* c*_amp_, as demonstrated in Fig. 20a. If *ε* > 0.032 mm, the vibration amplitude of the compound type chain will not be smaller than that of the base type one. Figure 20b shows the vibration locus of the compound type chain when *ε* = 0.05 mm, the existence of *ε* causes the chain pitch line to rise 0.1 mm higher in the vertical direction. Comparing with the basic vibration amplitudes, it can be seen from Fig. 20c that, when *ε* = 0.05 mm, the vibration amplitude of the most pitch points for the compound type chain is greater, and there is *c*_amp_ = − 16.76%. In conclusion, the existence of the inner meshing profile error will weaken the noise reduction in the low frequency range that caused by the inner meshing.

As for the vibration velocity of the chain, according to Eqs. (30) and (31), we can know that *ε* mainly affects the *X*-axis vibration velocity of the compound type HEVs coupler chain. Under the error, there is:

46 $$v_{X} (t) = \left\{ {\begin{array}{*{20}l} {\omega_{1} r_{{{\text{p1}}}} \cdot \cos (\varphi_{1} - \omega_{1} t), \, \frac{{\eta_{3} }}{{\omega_{1} }} + j\frac{{\varphi_{1} }}{{\omega_{1} }} \le t < \frac{{\eta_{4} }}{{\omega_{1} }}{ + }j\frac{{\varphi_{1} }}{{\omega_{1} }} \, } \hfill \\ {\omega_{1} (r_{{{\text{p1}}}} + \Delta_{{\text{p}}} ),\quad {\text{others}}} \hfill \\ \end{array} } \right.$$

Based on Eqs. (33) and (46), the RMS value of *v*_X_ is:

47 $$v^{\prime}_{{X{\text{R - er}}}} = \sqrt {\frac{{\int_{0}^{{\frac{{\eta_{3} }}{{\omega_{1} }}}} {[\omega_{1} (r_{{{\text{p1}}}} + \Delta_{{\text{p}}} )]^{2} {\text{d}}t + \int_{{\frac{{\eta_{4} }}{{\omega_{1} }}}}^{T} {[\omega_{1} (r_{{{\text{p1}}}} + \Delta_{{\text{p}}} )]^{2} {\text{d}}t} } + \int_{{\frac{{\eta_{3} }}{{\omega_{1} }}}}^{{\frac{{\eta_{4} }}{{\omega_{1} }}}} {[\omega_{1} r_{{{\text{p1}}}} \cdot \cos (\varphi_{1} - \omega_{1} t)]^{2} {\text{d}}t} }}{T}}$$

According to Eqs. (15) and (35), there is:

48 $$c_{vX} = \frac{{v_{{X{\text{R}}0}} - v^{\prime}_{{X{\text{R}}0 - {\text{er}}}} }}{{v_{{X{\text{R}}0}} }} \times 100\% = \frac{{v_{{X{\text{R}}0}} - v^{\prime}_{{X{\text{R}} - {\text{er}}}} + v^{\prime}_{X\min } }}{{v_{{X{\text{R}}0}} }} \times 100\%$$

We can obtain from Eqs. (33) and (47) that, under the error effect, the RMS value of *v*_*X*_ is increased for the compound type chain. Thus, based on Eq. (48), under the effect of *ε*, *c*_*vX*_ is decreased. Therefore, the inner meshing profile error may increase the medium frequency vibration and noise of the driven sprocket.

**Fig. 21:**
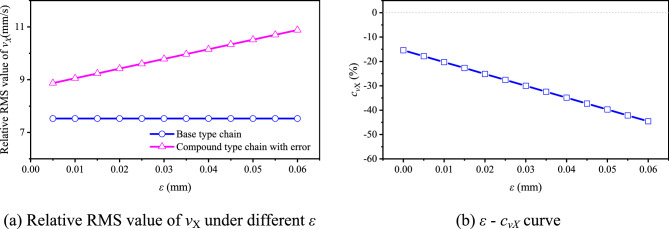
The influence of *ε* on the *X*-axis vibration velocity.

Based on the parameter values in Table 1, combining with Eqs. (33) and (47), we can obtain the relative RMS value of *v*_*X*_ under different *ε*, as Fig. 21a demonstrates. In Fig. 21a, the relative RMS value of *v*_*X*_ for the compound type chain with the error is increased with the increase of *ε*. It means that, the error *ε* will further increase the medium frequency vibration and noise in the horizontal direction. Such as in Fig. 21b, when *ε* = 0.05 mm, the percentage increase of the relative RMS value of *v*_*X*_ will be reached at 39.72%. In actual condition, the value of *ε* may be reached at 0.1 mm or even more. Therefore, under the influence of the error, the vibration velocity of the compound type HEVs coupler chain in the horizontal direction may be the main reason to cause the medium frequency noise.

If only considering the pitch line deviation, as for the vibration acceleration of the chain, according to Eqs. (37)–(39), the inner meshing profile error has no effect on that.

### Influence of meshing disorder

**Fig. 22:**
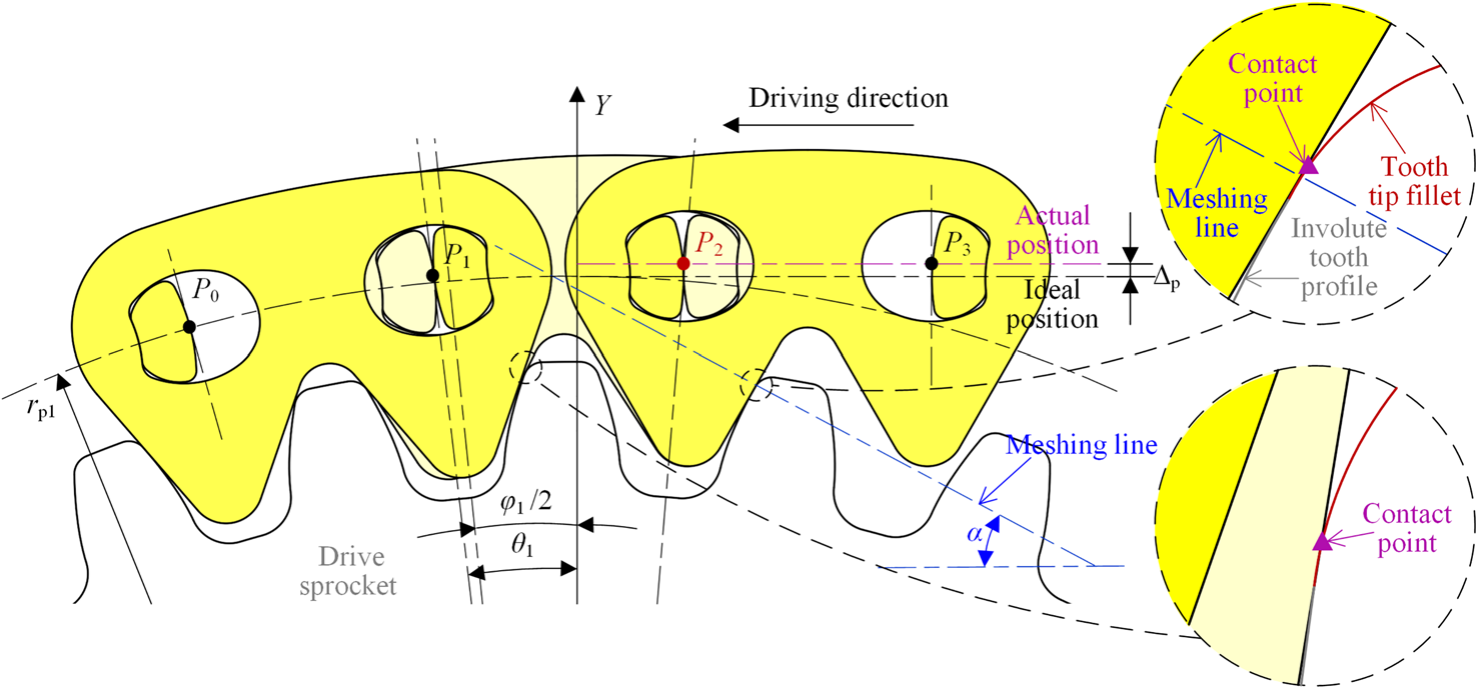
Disorder in meshing chain links under error.

In fact, the inner meshing profile error truly affects the vibration acceleration. The error not only deviates the chain pitch line from its ideal position, but also causes disorder in the meshing chain links, as Fig. 22 displays. When *θ*_1_ is close to and greater than *η*_3_, since the left inner meshing profile of link *P*_1_*P*_2_ will contact the tooth tip fillet of the drive sprocket, link *P*_0_*P*_1_ cannot be properly located on the sprocket. In addition, the error also makes the left side inner meshing profile of link *P*_2_*P*_3_ always in contact with the sprocket. Because the theoretical meshing point exceeds the range of the involute profile of the sprocket tooth, the left side inner meshing profile of link *P*_2_*P*_3_ can only contact with the tooth tip fillet of the sprocket, resulting in incorrect inner meshing. Due to the incorrect position of links *P*_0_*P*_1_ and *P*_2_*P*_3_, link *P*_1_*P*_2_ is also unable to properly mesh with the sprocket. Obviously, the meshing disorder of the compound type HEV coupler chain will increase the abnormal collisions between the chain and the sprockets. Thus, the vibration acceleration is no doubt to increase, and the noise in the high frequency range will increase too. It should be noted that the collisions caused by meshing disorder are chaotic and difficult to express with an equation, so no related equation will be provided here.

Based on the above analysis results, under the action of the inner meshing profile error, the noise of the base type HEVs coupler chain should be smaller than that of the compound type one at the beginning. Considering the wear resistance of the chain plate is worse than that of the rocker pins, the value of *ε* may be reduced by wear, thus the positive action of the inner meshing profile on the noise reduction will gradually appear. Therefore, we can assume that, after a certain period of running-in, the noise of the compound type chain will be smaller than that of the base type one.

## Dynamics simulation

**Fig. 23:**
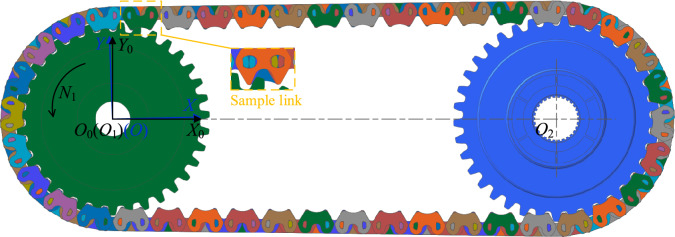
Multi-body dynamics model.

To verify the analysis results in “Basic vibration” and “Inner meshing profile influences” sections, we simulate the two types of HEVs coupler chain in CAE software *RecurDyn V9R3*(URL link: https://functionbay.com/en), both of them satisfy the values in Table 1, and the multi-body dynamics model is shown in Fig. 23. In the simulation, the rotation center *O*_1_ of the drive sprocket is fixed in the origin *O*_0_ of the absolute coordinate system *S*_0_(*X*_0_*O*_0_*Y*_0_), and the driven sprocket rotation center *O*_2_ is located on the *X*_0_-axis. *S*(*XOY*) is the coordinate system to analyze the vibration locus, and the *X*-axis is parallel to the tension side chain. In this section, we use the locus, velocity and acceleration of sample link to represent the vibration locus, velocity, and acceleration of the chain for analysis.

### Locus analysis

**Fig. 24:**
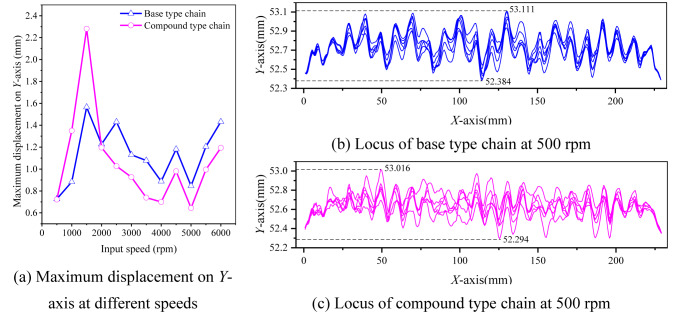
Simulation results about the locus of sample link.

Figure 24 shows the simulation locus of the two types of chain in the tension side. As shown in Fig. 24b,c, whether the base type chain or the compound type chain, the locus of sample link is different in each cycle. To make the analysis results more precise and reliable, more data needs to be considered. In this part, we use the maximum displacement over multiple cycles of sample link on the *Y*-axis to assess the low frequency vibration of the chain. For example, when input speed *N*_1_ is 500 rpm, as for the base type chain, the maximum displacement over multiple cycles on the *Y*-axis is equal to 53.111–52.384 = 0.727 mm (see Supplementary Table 2 for the complete dataset). As for the compound type chain, the value is 53.016–52.294 = 0.722 mm (see Supplementary Table 3 for the complete dataset). Based on the above calculation method, the maximum displacement over multiple cycles on the *Y*-axis for the two types of chain at different speeds can be calculated, as shown in Fig. 24a. From Fig. 24a, we can see that, except for the input speeds of 1000 rpm and 1500 rpm, the values of the maximum displacement on the *Y*-axis of the compound chain are smaller at other speeds (see Supplementary Table 1 for the complete dataset). Therefore, the inner meshing profile can improve the low frequency vibration of coupler chain to some extent at the most speeds.

It should be noted that in multi-body dynamics simulation, due to the influence of chain flexibility, the simulation results of different chain links are not the same, it means that the results have a certain degree of randomness. Therefore, when the input speed is 1000 rpm or 1500 rpm, even though the maximum displacement value of the base type chain is smaller, it does not mean that the low frequency vibration performance of the base type chain is better at these two speeds. We hold that the assessment of which type of chain has better low frequency vibration performance should consider the maximum displacement values at all speeds. Moreover, this method is also applicable to the analysis of velocity and acceleration.

### Velocity analysis

**Fig. 25:**
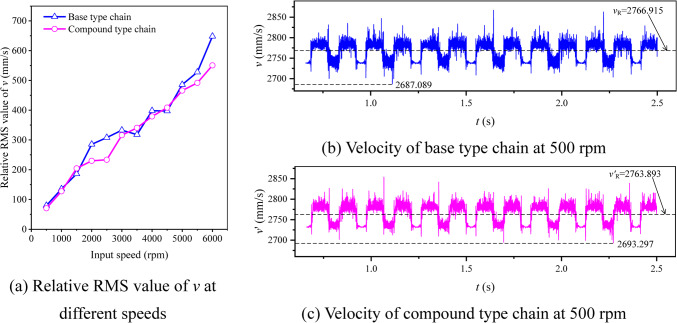
Simulation results about the velocity of sample link.

Figure 25 demonstrates the simulation results about the vibration velocity of the two types of chain. As Fig. 25b displays, when input speed is 500 rpm, the RMS value of the vibration velocity of the base type chain is 2766.915 mm/s, and the minimum value is 2687.089 mm/s, so the relative RMS value of the vibration velocity of the base type chain is 2766.915–2687.089 = 79.826 mm/s (see Supplementary Table 5 for the complete dataset). Similarly, based on Fig. 25c, we can obtain that the relative RMS value of the vibration velocity of the compound type chain is 2763.893–2693.297 = 70.596 mm/s (see Supplementary Table 6 for the complete dataset). Thus, we can obtain every relative RMS value of the vibration velocity of the two types of chain at all speeds, as Fig. 25a shown. It can be seen from Fig. 25a that, except for the input speeds of 1500 rpm, 3500 rpm, and 4500 rpm, the relative RMS values of the vibration velocity of the compound type chain are all smaller at other speeds (see Supplementary Table 4 for the complete dataset). It means that the inner meshing profile can reduce the vibration of coupler chain in the medium frequency range.

### Acceleration analysis

**Fig. 26:**
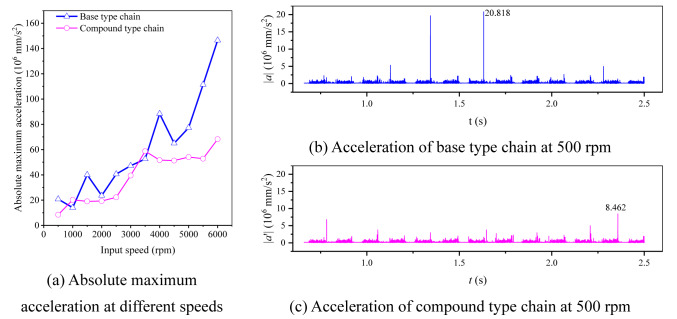
Simulation results about the acceleration of sample link.

Figure 26 displays the differences in absolute maximum acceleration between the two types of chain. From Fig. 26a, except for the input speeds of 1000 rpm and 3500 rpm, the values of the absolute maximum acceleration of the compound type chain are significantly lower than that of the base type chain at other speeds (see Supplementary Table 7 for the complete dataset). For example, as Fig. 26b,c, when *N*_1_ = 500 rpm, the absolute maximum acceleration of the compound type chain is only 8.462 × 10^6^ mm/s^2^, while that of the base type chain reaches 20.818 × 10^6^ mm/s^2^ (see Supplementary Table 8 and 9 for the complete dataset). It indicates that the inner meshing profile can improve the high frequency vibration of coupler chain.

It should be pointed out that the simulation results shown in Fig. 26 are not contradictory to the analysis results in “Influence on vibration acceleration” section. Although the analysis results in “Influence on vibration acceleration” section indicate that the maximum acceleration difference between the two types of chain should not be so significant, the maximum acceleration in “Influence on vibration acceleration” section is derived from static analysis, and its value is closer to the local maximum acceleration in the dynamics simulation results. Due to the abundance of data in multi-body dynamics simulations, it is difficult to extract and distinguish the local maximum acceleration data. Therefore, the global maximum acceleration from all data is used as a substitute here. Although the global maximum acceleration is not equal to the local maximum acceleration, their trends are the same.

Combining all the simulation data, the results show that only at an input speed of 1000 rpm, the base type chain exhibits potentially better vibration performance. At other speeds, the compound type chain shows superior vibration performance. Therefore, the results of the multi-body dynamics simulation are generally consistent with the theoretical results in “Basic vibration” and “Inner meshing profile influences” sections of this paper. Thus, it can be concluded that, without considering the error of the inner meshing profile, the vibration and noise performance of the compound type chain should be better.

## Noise and wear experiment

Because it is difficult to install the sensor on the chain, the real vibration of the chain link cannot be measured. Even though the sensor can be installed on the driven sprocket, measuring the vibration of the driven sprocket is meaningless because the vibration of the sprocket is not equal to that of the chain^19,22,28^. In this section, the wear experiment is used to assist in verifying the correctness of the vibration analysis and to analyze the influence of the inner meshing profile on noise.

Although the A-weighted sound pressure level is closer to human auditory perception, its modifications result in a lesser correlation between vibration compared to the Z-weighted sound pressure level. Therefore, in this section, we will use the Z-weighted sound pressure level for analysis. It should be noted that although we have used the Z-weighted sound pressure level, the results obtained under the Z-weighting are quite similar to those obtained under the A-weighting.

### Equipment and method

**Fig. 27:**
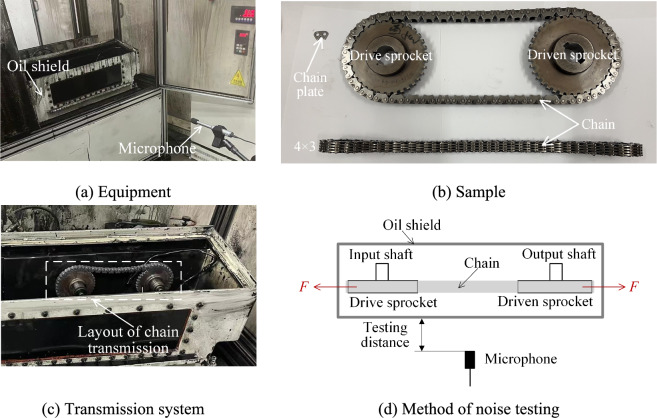
Method and equipment.

In general chain noise research, no load is applied to the chain transmission system. However, to verify the applicability of the theory proposed in this paper, the noise tests are conducted on a wear test bench that allows for load application. The bench is independently developed by Qingdao Choho Industrial Co., Ltd. As Fig. 27a shows, the test bench is placed in a completely closed semi-anechoic chamber, and a professional ambient acoustic microphone(MINIDSP UMIK-1) is used. The projection of the microphone on the oil shield coincides with the center of that surface of the oil shield. The sampling frequency of the microphone is 48,000 Hz, and the sensitivity of that is 1 V/Pa. The lubrication method of the test bench is oil injection lubrication, and the chain transmission system is running in the oil shield, as Fig. 27c displays. Figure 27b shows the test sample, in this paper, we manufactured two types of HEVs coupler chain, both of them satisfy the values in Table 1, and the chain form is 4 × 3. Figure 27d shows the method of the noise testing, during the experiment, the test bench applies input speed and load *F* to the chain drive system, the input speed is 500–6000 rpm, and the load *F* is 0, 500 N, 1000 N, 1500 N respectively. The testing distance is the distance between the geometric center of the surface of the oil shield and the microphone, and the testing time is 30 s.

### Noise before running-in

#### Noise level analysis

**Fig. 28:**
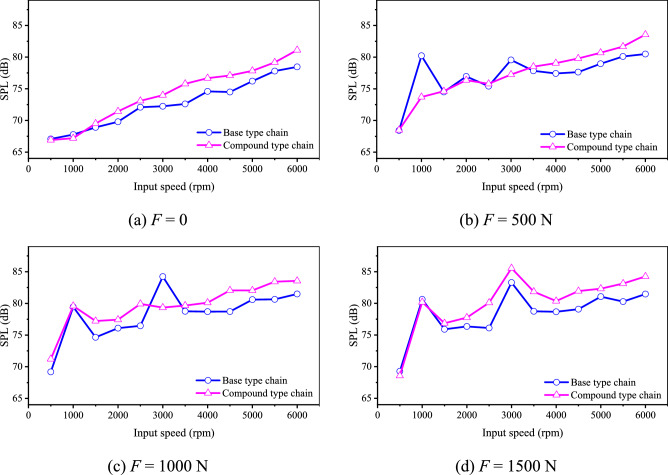
Noise before running-in (Detailed data can be found in Supplementary Table 10).

If we directly measure the running noise of the freshly machined chain, the results are shown in Fig. 28. It can be seen from Fig. 28 that:

When the load is 0, 1000 N, and 1500 N, the noise level of the compound type chain is generally greater than that of the base type chain at most speeds. When the load is 500 N, within the speed range of 1000 rpm to 3000 rpm, the noise level of the base type chain is greater than or shows an increasing trend compared to the noise level of the compound type chain. Overall, under different loads, the noise performance of the base type chain is better at most speeds.

At the highest speed (6000 rpm), compared with the base type chain, the noise level of the compound type chain is increased by 2.66 dB, 3.06 dB, 2.07 dB, and 2.78 dB respectively when the load is equal to 0, 500 N, 1000 N, and 1500 N respectively. Obviously, the situation is contrary to the results in “Inner meshing profile influences” and “Dynamics simulation” sections. It means that the advantages of the inner meshing profile for noise reduction are difficult to appear if the chain is without running-in, which may be caused by the error of the inner meshing profile. It should be noted that, because the chain is without running-in, the noise level of the chain may not be strictly increased with the increase of the input speed when the load *F* is not equal to zero.

#### Spectrum distribution analysis

**Fig. 29:**
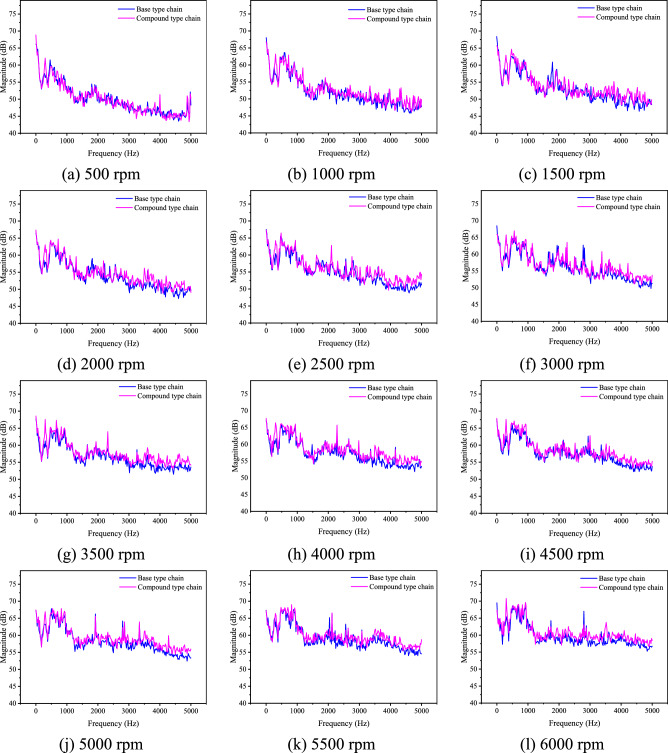
Spectrum distribution of noise before running-in when *F* = 0 (Detailed data can be found in Supplementary Table 11).

To further verify the analysis results in “Error influence” section, the spectrum distribution of the noise before running-in should be discussed. Figure 29 shows comparison about the noise spectrum distribution at all speeds when *F* = 0.

It can be obtained from Fig. 29 that the magnitude of the compound type chain is relatively high in the frequency range of 0 to 2000 Hz, especially in the range of 180 to 400 Hz. When frequency is bigger than 2000 Hz, the magnitude of the compound type chain is visibly greater than that of the base type chain. It means that the vibration and noise of the compound type HEVs coupler chain is relatively high across the entire frequency range, which is consistent with the analysis results in “Error influence” section.

In addition, we can also obtain from Fig. 29 that for both types of the chain, the magnitude in the mid-low frequency range (0–1500 Hz) is relatively larger compared to other frequency ranges. Therefore, it is reasonable to study the noise of HEVs coupler chain based on the vibration caused by the polygonal action. When the load *F* changes, the relationship of the noise characteristics of the two types of chain before running-in is not significantly changed, and this paper will not elaborate further on this.

According to general logical reasoning, if the noise of the compound type chain is larger in the high frequency range, it indicates that the vibration of the chain in the high frequency range is also larger, and its expected wear degree is greater. After running-in, theoretically, the high frequency noise difference between the compound type chain and the base type chain should be further increased. However, after running-in process, if the wear degree of the compound type chain is less and the high frequency noise is lower, the only reason is that the compound type chain has better noise performance and stronger wear resistance, thus the wear mainly occurs on the inner meshing profile rather than the rocker pin. Moreover, it can also prove that the high-level noise of the compound type chain before running-in is mainly caused by the inner meshing profile error, thus the correctness of the results from “Basic vibration” to “Error influence”sections can be verified.

### Wear analysis

**Fig. 30:**
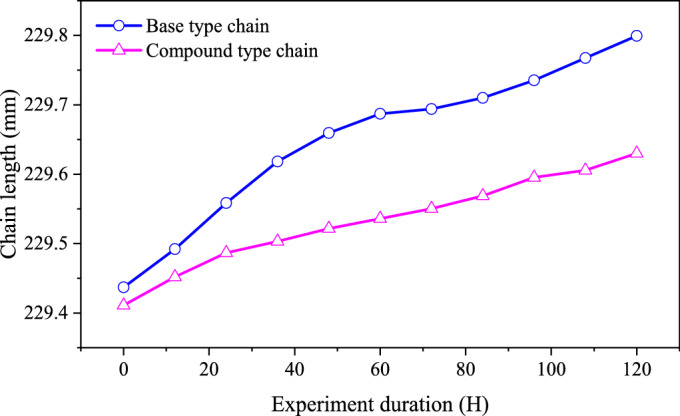
Measuring values of the chain length (Detailed data can be found in Supplementary Table 12).

In this paper, we regard the wear experiment as the complete running-in process of the chain. In the wear experiment, we set the experiment duration to 120 h, measuring every 12 h, with a load *F* = 500 N and an input speed of 5000 rpm. Without damaging the chain, the system center distance is usually used to represent the chain length. The greater the measuring value of the chain length, the poorer the wear resistance of the chain^21^. Figure 30 demonstrates the measuring values of the chain length for two types of chains in the wear experiment.

From Fig. 30, after running-in, the chain length of the base type chain is changed from 229.437 to 229.800 mm, the percentage increase is 0.158%. The length of the compound chain is changed from 229.411 to 229.629 mm, the percentage increase is 0.095%. Compared with the base type chain, the wear resistance of the compound type chain is improved by 39.87%. Generally, the elongation of the chain after running-in is caused by the wear of the pin^26^. Therefore, we can obtain that Eqs. (40) and (41) are correct, and the higher vibration of the compound type chain in the high frequency range mainly causes the wear of the inner meshing profile, instead of the wear of the rocker pin. This result is an important premise that validates the correctness of the analysis in this paper.

### Noise after running-in

#### Noise level analysis

**Fig. 31:**
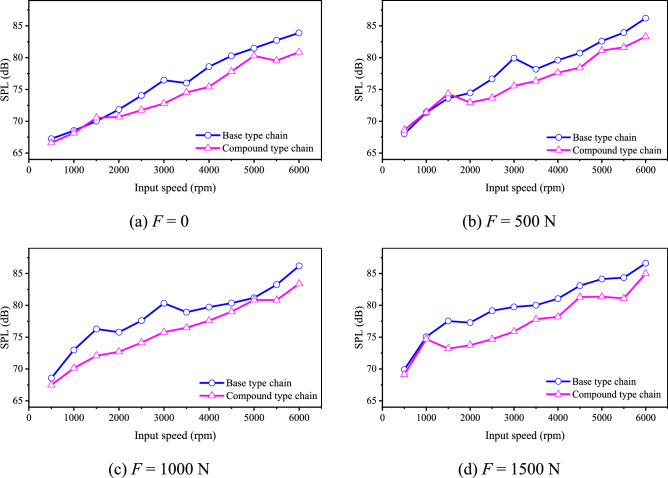
Chain noise after running-in (Detailed data can be found in Supplementary Table 13).

Figure 31 shows the noise level of the chain after running-in, we can obtain: Under most conditions, the noise level of the compound type chain is obviously smaller. At the highest speed (6000 rpm), compared with the base type chain, the noise level of the compound type chain is reduced by 3.02 dB, 2.9 dB, 2.8 dB, 1.62 dB respectively when the load is equal to 0, 500 N, 1000 N, and 1500 N respectively. Combined with the results in “Wear analysis” section, we can obtain that with the time going on, due to the error of the inner meshing profile is eliminated by wear, the inner meshing profile can markedly improve the noise performance of HEVs coupler chain.

In addition, after running-in, the noise level of the chain will be strictly increased with the increase of the input speed. Therefore, the noise level at the highest input speed is also the maximum noise level.

#### Spectrum distribution analysis

**Fig. 32:**
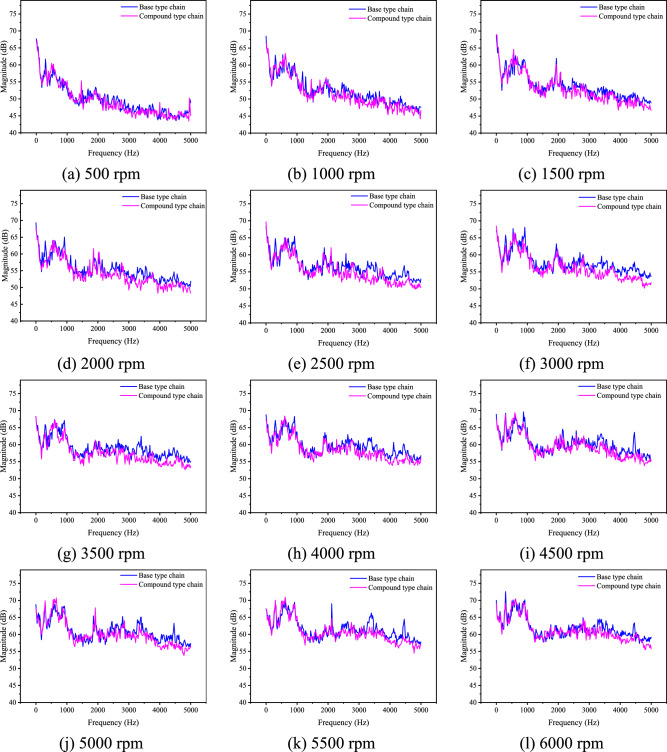
Spectrum distribution of noise after running-in when *F* = 0 (Detailed data can be found in Supplementary Table 14).

To further verify the noise characteristics of the compound type chain, the comparison analysis of the spectrum distribution of the noise after running-in is necessary. Figure 32 shows comparison about the noise spectrum distribution after running-in at all speeds when *F* = 0.

According to Fig. 32, we can know that the magnitude of the compound type chain is lightly smaller than that of the base type one in the frequency range of 0 to 2000 Hz. When in the frequency range of 2000 to 5000 Hz, the magnitude of the compound type chain is significantly less than that of the base type one, which is not completely consistent with the conclusions in “Influence on vibration acceleration” section. In this paper, we believe that the reason for the significant difference in high frequency noise is the greater wear degree of the base type chain. As a result, the noise performance of the compound type chain is proved to be better, and the analysis results in “Basic vibration” to “Dynamics simulation” sections are proved to be true. Similar as the relationship of the noise characteristics of the two types of chain before running-in, that after running-in will not be significantly changed with the load *F*.

#### Influence of running-in on noise

**Fig. 33:**
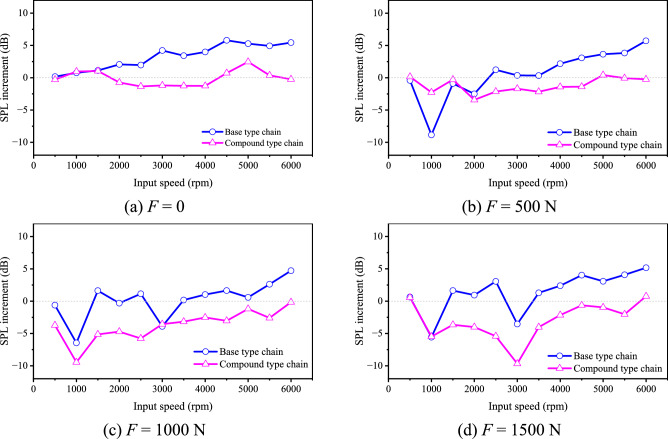
Difference of noise level (Detailed data can be found in Supplementary Table 15).

To analyze the influence of the chain running-in on noise, we calculate the difference between the noise level before running-in and that after running-in for the two types of chain, the results are demonstrated in Fig. 33.

It can be seen from Fig. 33 that the noise level of the compound type chain at most input speeds is reduced after running-in, and that of the base type chain at most input speeds is increased. As for the base type chain, the average noise level increment under 0, 500 N, 1000 N, 1500 N is 3.26 dB, 0.65 dB, 0.19 dB, and 1.43 dB respectively. As for the compound type chain, the average noise level increment under 0, 500 N, 1000 N, 1500 N is − 0.07 dB, − 1.21 dB, − 3.75 dB, and − 3.06 dB respectively. The phenomenon might be caused by the error of the inner meshing profile and the higher wear resistance of the compound type chain.

In conclusion, with time going on, the increase of the noise for the compound type chain will be smaller than that for the base type chain, which is consistent with the analysis results in “Influence on vibration acceleration” section.

## Conclusions

1. The inner meshing profile can reduce the vibration of HEVs coupler chain, and it also has the potential to reduce the chain noise. Based on the specific example in this paper, the inner meshing profile can reduce the vibration amplitude, the RMS value of *Y*-axis vibration velocity, and the absolute maximum values of the *X*-axis/*Y*-axis vibration acceleration by 31.25%, 35.56%, 4.39%, and 0.05% respectively. It means that the inner meshing profile can reduce the vibration of the chain in three frequency ranges: low, medium and high, thus the noise in the relative frequency ranges have the potential to be reduced.
2. Under the effect of the error of the inner meshing profile, the noise level of the chain with the inner meshing profile may be greater than that of the classical chain at the beginning. Firstly, the error will enhance the chain pitch line to increase the vibration amplitude and velocity. For example, when the error is equal to 0.05 mm, the vibration amplitude is increased by 16.76%, and the relative RMS value of *X*-axis vibration velocity is increased by 39.72%. Secondly, the error will cause the meshing disorder to increase the meshing impact, resulting in the increase of vibration in the high frequency range. In the experiment, before running-in, the noise level of the compound type chain at the highest speed is 2.07–3.06 dB greater than that of the base type chain, and the high frequency noise of the compound type chain is higher. This is the main reason to cause the controversy about the influence of the inner meshing profile on noise.
3. Even though the high frequency noise of the HEVs coupler chain with the inner meshing profile is higher, the wear caused by high frequency vibration mainly occurs on the inner meshing profile rather than rocker pin. Because the error can be reduced by wear, in the stable running state, the noise level of HEVs coupler chain with the inner meshing profile is smaller than that of the classical one. In the experiment, after running-in, when the load is equal to 0, 500 N, 1000 N, 1500 N respectively, the maximum noise level of the compound type chain is 3.02 dB, 2.9 dB, 2.8 dB, 1.62 dB respectively smaller than that of the base type chain. This is the main reason that the inner meshing profile can improve the noise performance of HEVs chain.
4. Due to the higher wear resistance, the noise increment of the chain with the inner meshing profile is smaller than that of the classical one. In the experiment, the wear resistance of the compound type chain is 39.87% better than that of the base type one. Compared with the noise before running-in, the average noise level of the compound type chain after running-in is reduced by 0.07–3.75 dB, but the percentage reduction for the base type one is − 3.16 to − 0.19 dB. Moreover, after running-in, the high frequency noise of the chain with the inner meshing profile is significantly less than that of the classical one. This is a secondary reason that the inner meshing profile can improve the noise performance of HEVs chain.

Since the simulation and experimental results are consistent with the theory in this paper, it indicates that the noise of the coupler chain is more influenced by the flexible vibration of the chain, which depends on the meshing between the chain and the sprocket, rather than the rigid vibration of its constituent components. Because there is dual effect of the inner meshing profile on the chain noise, when studying the noise performance of HEVs coupler chain, we should pay more attention to whether the chain sample has been run in.

## Supplementary Information

Supplementary Information 1.

Supplementary Information 2.

Supplementary Information 3.

Supplementary Information 4.

Supplementary Information 5.

Supplementary Information 6.

Supplementary Information 7.

Supplementary Information 8.

Supplementary Information 9.

Supplementary Information 10.

Supplementary Information 11.

Supplementary Information 12.

Supplementary Information 13.

Supplementary Information 14.

Supplementary Information 15.

## Data Availability

All data generated or analyzed during this study are included in this published article [and its supplementary information files].
